# Pharmacological Evaluation of *Angelica keiskei* Extract: Molecular Interaction Analysis in Hepatocellular Carcinoma

**DOI:** 10.3390/cimb47060401

**Published:** 2025-05-29

**Authors:** Alka Ashok Singh, Minseok Song, Gun-Do Kim

**Affiliations:** 1 Department of Life Sciences, Yeungnam University, Gyeongsan 38541, Republic of Korea; 2 Laboratory of Cell Signaling, Department of Microbiology, College of Natural Science, Pukyong National University, 45 Yongso-ro, Nam-Gu, Busan 48513, Republic of Korea

**Keywords:** *Angelica keiskei*, anticancer, hepatocellular carcinoma, antibody apoptotic array, principal component analysis (PCA), HepG2 cells

## Abstract

Hepatocellular carcinoma (HCC), the most prevalent primary liver cancer, is the most significant cause of cancer-related death globally, with limited treatment options, including surgical resection, liver transplantation, ablation, chemoembolization, immunotherapy, and radiation. *Angelica keiskei*, a plant that is rich in chalcones and flavonoids, has demonstrated interesting anticancer properties. This study assesses the pharmacological effects of *Angelica keiskei* extract on HepG2 cells in order to investigate its efficacy as a therapeutic intervention for HCC. Using in vitro cell culture models, HepG2 cells were treated with different doses of the extract, and its cytotoxic and apoptotic effects were studied. GC-MS analysis revealed the presence of several bioactive compounds, including DDMP, which are likely involved in the observed effects. The MTT assay revealed a considerable, dose-dependent reduction in cell viability, with higher dosages causing notable morphological alterations. An antibody apoptotic array indicated significant changes in apoptotic proteins, specifically IGFBP1, BAD, and Bid. Cluster heatmaps, volcano plots, STRING analysis, Voom-mean variance trends, Glimma plots, and PCA were used to obtain an understanding of the molecular interactions involved. These results suggest that *Angelica keiskei* extract can cause apoptosis in HepG2 cells, with DDMP appearing as a potentially significant contributor. However, more experimental validation is required to determine the precise molecular mechanisms driving these favorable effects and their clinical implications in HCC.

## 1. Introduction

Cancer remains a global epidemic with an ever-increasing death rate, owing to a range of factors such as demographic shifts, rising life expectancy, and changes in the incidence and distribution of important risk factors. Hepatocellular carcinoma (HCC) is a lethal malignancy that necessitates early diagnosis and treatment. Medical research discoveries are increasing our understanding of this ailment, paving the way for more effective treatment alternatives.

In vitro cell culture, along with in silico models, are more cost-effective and accurate alternatives to animal research for determining drug toxicity and cell survival. This study utilized an in vitro approach along with an in-silico model to evaluate the anticancer activity of *Angelica keiskei* against HepG2 human liver cancer cells. According to a review [1], most liver tumors metastasize elsewhere in the body. HCC is the most common primary liver cancer and the leading cause of cancer-related deaths worldwide [2]. Indeed, it is the fourth leading cause of cancer-related deaths worldwide, underscoring its substantial impact on cancer-related mortality specifically attributable to cancer [3]. HCC accounts for 80–90% of primary liver cancers, making it the most frequent [4]. In the United States, primary liver cancer accounts for approximately 2% of all liver cancers. In some emerging regions, liver cancer accounts for up to 50% of all cancer cases, with hepatocellular carcinoma (HCC) being the most common type. Men are 2.4 times more likely to be diagnosed with HCC than women. The average 5-year survival rate for HCC is approximately < 15% [5]. In 2018, liver cancer was the sixth most frequently diagnosed cancer and the fourth major cause of cancer-related mortality. Compared with other malignancies, 841,000 (4.7 percent) new liver cancers were diagnosed in 2018, with 782,000 (8.2%) deaths [6]. The regions of Northern Africa, Southeast Asia, and Eastern Asia have been reported to have the highest incidence rates of liver cancer [7]. Recent studies on human hepatocytes have indicated that naturally occurring compounds and herbs play crucial roles as chemo-preventive agents in cellular and molecular biology [5].

Commonly known as Ashitaba in Japan [8], *Angelica keiskei* [9] (Umbelliferae) [10] belongs to the carrot family and is celebrated for its abundant nutrients and minerals. Its amino acids and minerals are recognized as protective mechanisms against a variety of major illnesses. *Angelica* keiskei is an herbal plant that grows primarily on Japan’s Pacific coast and is used in a variety of foods. Tea, bread, wine, and cosmetics all contain the aerial component [11]. In Korea, the plant is known as ‘Myeong-I1 Yeob’, while in Japan, it is known as Ashitaba. Both names refer to the year’s leaf [12]. Various biological actions of crude extracts and constituents of *Angelica keiskei* have been demonstrated, including anticancer effects. Owing to its bioactive substances, such as chalcone, coumarin, and phytochemicals, *Angelica keiskei* has pharmacological benefits, including liver protection, nerve protection, lipid-lowering, and anticancer properties [13]. It contains many active compounds, such as chalcones and coumarins. Two major chalcone compounds, xanthoangelol and 4-hydroxyderricin, exhibit antitumor activities [14]. The cytotoxicity of six chalcones from *Angelica keiskei* and two from *Humulus lupulus* L. (hop) was evaluated in IMR-32 and NB-39 human neuroblastoma cell lines and has been shown to cause apoptosis [9]. Botanical properties, phytochemical research, and biological studies on the extracts and pure constituents of *Angelica keiskei* are summarized in this article.

The antitumor effects of *Angelica keiskei* have been reported in various cancer cell lines via different signaling pathways. The extract induces apoptosis in various human cancer cell lines, including breast cancer cells (MDA-MB-231) [15]. After surgical removal of primary tumors from an active ethyl acetate fraction of 50% ethanol extract, *Angelica keiskei* powder containing 4-hydroxyderricin was identified as an active compound that inhibits tumor growth in Lewis lung carcinoma (LLC)-bearing mice, prolongs survival time, and suppresses metastasis to the lungs [16]. Another clinical study showed that after *Angelica keiskei* supplementation, neutrophil infiltration of infected tissues in mice was decreased [17]. Cell growth was inhibited, and cytotoxicity was observed in human oral squamous cell carcinoma cells, proving the efficacy of *Angelica keiskei* [18]. Hepatocellular necrosis was mitigated, and serum bile acid and bilirubin levels decreased in bile duct ligated mice when *Angelica keiskei* extract (AKE) (200 or 500 mg/kg) was administered orally [19]. There were relatively few differences between untreated HEK293 and *Angelica keiskei*-treated HEK293 cell lines, as reported in a recent study, suggesting that *Angelica keiskei* does not damage normal cells [20]. In addition, studies have reported the antioxidant, anti-depressant, anti-viral, antithrombotic, anti-hypertensive, anti-inflammatory, and anti-allergic activities of *Angelica keiskei* derivatives and crude materials from other parts of the plant [11]. Although many studies have examined the effects of *Angelica keiskei* on various targets by isolating its component chemicals, 2,3-Dihydro-3,5-dihydroxy-6-methyl-4H-pyran-4-one (DDMP) has not yet been employed to improve the anticancer efficacy against HepG2 cells. Antitumor effects of *Angelica keiskei* have been reported in various cancer cell lines. The anticancer effects of *Angelica keiskei* on human liver cancer HepG2 cells have not yet been thoroughly elucidated. This study uses the “Human Apoptosis G1” series assay, as well as molecular docking analysis, to demonstrate *Angelica keiskei*’s anticancer activity and identify its safety efficacy.

## 2. Materials and Methods

### 2.1. Reagents

Crude *Angelica keiskei* was obtained from www.handsherb.co.kr, Gyeonggi-do, Republic of Korea and the HepG2 cell line was purchased from ATCC (Manassas, VA, USA). A Minimum Essential Medium (MEM Gibco, Thermo Fisher Scientific (Waltham, MA, USA)) was used to treat the HepG2 cells.

### 2.2. Crude Extraction (Sample Treatment and Subcritical Water Extraction Conditions)

The roots and leaves of dried *Angelica keiskei* were ground up in a blender and sieved through a 450 µm mesh screen before being processed in a batch subcritical water reactor (1 L). Subcritical water experiments were conducted using 100% distilled water at temperatures ranging from 140 °C to 170 °C, with a 10 °C spike between each condition. Except for one state (160 °C, 45 mg/mL), the pressure, reaction time, and solid/liquid ratio were all held constant at 3 MPa, 10 min, and 30 mg/mL, respectively, to examine the possible effect of altering the solid/liquid ratio. In a unique setting at 160 °C, 50% ethanol was used instead of 100% water to improve the extraction efficiency of less polar chemicals. Below is a Table 1 listing the treatments and their respective acronyms [21].

### 2.3. GC-MS

Gas chromatography–mass spectrometry (GC-MS) analysis was performed using a GCMS-QP2010 Ultra system (Shimadzu, Kyoto, Japan) equipped with a DB-5MS Ultra capillary column (30 m × 0.25 mm I.D., 0.25 µm film thickness) to identify the compounds. The substance was dissolved in water, and methanol was added for further dilution. After centrifugation, the supernatant was collected to separate the precipitated fractions. Detailed GC-MS chromatograms and identified compound profiles are provided in the Supplementary Materials.

### 2.4. Cell Culture

The cells were cultivated in MEM medium under standard incubator conditions (37 °C, 5% CO_2_, and 95% humidity). HepG2 cells were grown to 85% confluence and treated with *Angelica keiskei* in a time- and dose-dependent manner from passages one to four to determine the anticancer efficacy of the compound.

### 2.5. Solution Preparation

The *Angelica keiskei* sample was dissolved in distilled water, and a stock was prepared at a 1 mg/mL concentration. Then, a standard solution was prepared with various dosages, which ranged from 0 to 400 µg/mL, and treated.

### 2.6. MTT Assay

HepG2 cells were grown in MEM until they reached approximately 85% confluence, then seeded in 96-well plates and incubated for 24 h under standard conditions. After removing the old medium, cells were treated with various concentrations of *Angelica keiskei* extract in fresh MEM and incubated for another 24 h. Following treatment, the medium was replaced with MEM containing 10 µL of EZ-Cytox reagent (WST-1; Daeil Lab Services, Seoul, Republic of Korea) per well. A control well without *Angelica keiskei* was included. The plates were incubated at 37 °C for 2 h in the dark, then gently shaken for 5 s before reading absorbance at 460 nm using an ELISA microplate reader. The data were used to assess cell viability and generate graphical representations of the results.

### 2.7. Human Apoptotic Array Screening

A human apoptotic array was used to determine the molecular pathways involved in *Angelica keiskei*-treated HepG2 cells.

#### Sample Preparation

The protein was extracted using protein 1X Cell Lysis Buffer, which was thoroughly diluted 2-fold with ddH_2_O before use. It contained a 1% protease inhibitor cocktail and a 1% phosphatase inhibitor cocktail (Sigma, St. Louis, MO, USA). Freezing at −80 degrees for at least 20 min after protein extraction is recommended. The material was then centrifuged for 20 min to defrost. This procedure was repeated at least three times. The samples were then refined using the steps outlined above (100 µL of sample volume is required for each array. This matrix effect can be avoided. For bodily fluids like serum, plasma, and cell culture medium, a minimum of a 2 × dilution is suggested or 500 µg/mL–1 mg/mL (diluted 5- to 10-fold to reduce detergent effects)). The BCA protein assay kit (Pierce, Rockford, IL, USA) was used with a Multi-Skan FC (Thermo, Waltham, MA, USA) to analyze the concentrated sample for protein concentration. The UV spectra confirmed that the purified sample was pristine. Protein samples were quantified using the BCA assay and normalized to 500 µg/mL before loading onto the antibody array. This ensured equal protein input across all samples.

### 2.8. Antibody Array Assay

The following methodology is involved in performing the antibody array assay.

#### 2.8.1. Blocking and Incubation

Blocking slides required the addition of 100 µL of 1X Blocking Buffer into each well, followed by an incubation period of 30 min at room temperature. After the buffer had been removed from each well, 100 µL of each sample was added to the wells corresponding to those samples. The arrays were incubated at room temperature for two hours. After removal from the wells, the samples were washed five times for two minutes each in 150 µL of 1X Wash Buffer I at room temperature with gentle rocking. The 20× Wash Buffer I was diluted with H_2_O. Each well had the 1× Wash Buffer I drained out of it and washed twice for two minutes with 150 µL of 1× Wash Buffer II while gently rocking at room temperature. After each wash, the wash buffer was effectively removed and diluted with Wash Buffer II (20×) with H_2_O.

#### 2.8.2. Incubation with Biotinylated Antibody Cocktail and Wash

After incubating at room temperature for 2 h, 70 µL of the detection antibody cocktail was applied to each well. After removing the samples from each well, they were washed at room temperature with moderate rocking five times using 150 microliters of 1× Wash Buffer I for two minutes each, followed by two washes using 150 microliters of 1× Wash Buffer II for two minutes each. The wash buffer was removed during each phase of the washing process.

#### 2.8.3. Incubation with Cy3 Equivalent Dye-Streptavidin and Wash

The 70 µL of dye-conjugated streptavidin conjugated with the Cy3 equivalent was applied to each well. The slide chamber was shielded from light so that it would not be exposed to light and would be incubated in a dark environment. The device was then maintained at room temperature for two hours. After removing the samples from each well, they were washed at room temperature two times, each time for two minutes, with 150 microliters of 1× Wash Buffer I in a gently rocking motion. After gently removing the slide from the gasket, the device was disassembled by pressing the clips on the slide side outward and removing it. The slide was placed in a Slide Washer/Dryer (a 4-slide holder/centrifuge tube), and enough 1× Wash Buffer I was added (about 30 mL) to cover the whole slide and then gently shaken at room temperature for 10 min (2 times). The slides were washed with 1× Wash Buffer II (approximately 30 mL) and gently shaken at room temperature for ten minutes. This was performed after washing with decanted Buffer I. The slides were cleaned with distilled water prior to examination.

#### 2.8.4. Data Analysis

A GenePix 4100A scanner (Axon Instruments, Union City, NJ, USA) was used for slide scanning. Before scanning the slides, we ensured that they were completely dry and scanned them within 24–48 h. The slides were scanned using an ideal laser power setting, a PMT, and a resolution of 10 um. The scanned images were gridded and quantified using GenePix software version 7.0 (Axon Instrument, Union City, NJ, USA). UniProt D.B. was used to annotate data pertaining to protein information. Data mining and graphic visualization were performed using the ExDEGA software (http://www.e-biogen.com, E-Biogen Inc., Seoul, Republic of Korea).

### 2.9. Apoptotic Array Data Analysis

This comprehensive analysis included various methodologies designed to understand and analyze apoptotic array data. This study uses techniques such as principal component analysis (PCA) and voice transformation to identify the underlying patterns and decrease data complexity. Glimma (version 2.6.0) provides an interactive visualization to explore differential expression results, whereas cluster heatmap analysis identifies co-expression relationships among apoptotic genes. Volcano scatter plots showed significant gene changes, whereas protein/protein interaction network analysis revealed changes in the interactome, leading to a deeper understanding of apoptotic processes.

#### 2.9.1. Cluster Heat Map

Microarray technology dominated high-throughput gene expression profiling for more than a decade before the debut of RNA-seq technology. Regardless of the high-throughput gene expression profiling assay employed, heat mapping is one of the most common methods for presenting gene expression data. A heat map is a graphical representation of data in which the individual values of a matrix are represented by colors [22]. Cluster analysis is another prominent method used for studying gene expression [23]. In the present work, we have utilized heatmap for visualization and quality control of the data using the “heatmap” function in R.

#### 2.9.2. Volcano Scatter Plot

A volcano scatter plot is another graphical representation that is frequently used in biological data analysis, particularly with high-throughput omics data such as gene expression data from RNA-seq or microarray research. Volcano plots have been used to discover genes with significantly varying expression levels across experimental settings or groups. Genes in the higher portions of the plot (above a particular level of statistical significance) are frequently regarded as potential candidates for future studies because they reflect genes with significant changes in expression that are statistically robust. Volcano scatter plots were generated using R software v4. The web app VolcaNoseR allows users to generate, examine, label, and share volcano plots (https://huygens.science.uva.nl/VolcaNoseR, accessed on 26 May 2025). A web tool may build a personalized, interactive volcano plot by retrieving data from an online source and incorporating user-defined options [24].

#### 2.9.3. Protein/Protein Interaction Network Investigation

Specialized tools continue to develop rapidly in all areas of network creation and analysis. Functional characterization of the proteins in the interaction network was performed to determine their biological roles and importance. Cancer is a complex disease, and many genes involved in cancer development have been identified. Studying cancer proteins within the human protein/protein interaction network could offer crucial insights into cancer and other complex diseases, bypassing the need to analyze individual genes or loci [25].

#### 2.9.4. Principle Component Analysis

Large amounts of genetic data are produced daily because of advances in sequencing technologies. However, without a thorough study, these data would not have been valuable. Several analytical methods, including pattern extraction, prediction, clustering, and classification, are crucial for drawing insightful conclusions from unprocessed data. These strategies rely on selecting relevant data properties to ensure accurate findings. However, the high dimensionality of bioinformatics data, with hundreds of features, can render machine-learning techniques less effective. To decrease the number of attributes and facilitate a more efficient analysis of bioinformatics data, dimensionality reduction techniques such as PCA are used [26]. In the present study, apoptotic array experiments on HepG2 cells treated with crude extract were performed to minimize the dimensionality of various datasets using PCA. PCA was applied to reduce the dimensionality of the dataset while preserving most of the variability found in the original data. This makes it easier to explore and visualize data in a lower-dimensional space.

#### 2.9.5. Glimma Plot

The Glimma R package was used to generate personalized interactive graphics for differential expression (DE) analysis. Glimma is accomplished with “Interactive Graphics” and “Limma” packages in R. Basic plots provided by Glimma include the mean-difference (MD) plot, which can be obtained by glMDPlot, and the multidimensional scaling (MDS) plot, which can be accessed via the glMDSPlot function. Using the eigenvalue decomposition of Euclidean distances computed from gene expression profiles, the MDS plot arranges the samples using an unsupervised clustering technique [25].

#### 2.9.6. Voom

The “Voom” function in the Limma R package is used to robustly estimate the mean-variance relationship and assign precision weights to each normalized observation. These weighted log counts per million were then used in the Limma analysis pipeline, making the RNA-seq data compatible with methods developed for microarrays and facilitating comparable analysis [27]. 

The R Limma package was employed for Voom transformation, using log2-counts per million (logCPM) to stabilize the variance and prepare the data for linear modeling. Antibody array assay is a prominent genomic technique used in biological research. DNA microarrays have long been the gold standard for genome-wide gene expression studies and are supported by a wealth of statistical techniques for analyzing microarray intensity data [28]. This section covers the techniques used for the analysis of differential expression. The first approach is referred to as limma-trend, and the second as Voom, which stands for “variance modeling at the observational level”. Whereas Voom applies the mean-variance connection at the individual observation level, Limma-trend applies it at the gene level [27]. Linear modeling was performed after Voom transformation and variance modeling to identify differentially expressed genes (DEGs) between experimental conditions or groups of interest. The significance of differential expression was evaluated using Limma’s moderated *t*-test.

### 2.10. Molecular Docking Analysis

The ligand 2,3-Dihydro-3,5-dihydroxy-6-methyl-4H-pyran-2-one was docked into the active site of the insulin growth factor-binding protein to explore the inhibition process. The AutoDock Vina v.1.5.7 model with the lowest binding energy was chosen after docking. Binding pockets were identified using the prank web tool, ensuring all binding-site residues were contained within the specified grid box. Molecular interactions within the complexes were analyzed using the Discovery Studio Visualizer tool.

### 2.11. Statistical Analysis

All experiments were conducted, and data were analyzed using one-way analysis of variance (ANOVA) and presented as mean ± standard deviation (SD). Statistical significance was defined as *p* < 0.05. GraphPad Prism software (version 5.1) was employed for all statistical analyses.

## 3. Results and Discussion

### 3.1. Extraction of Angelica keiskei and Compound Identification Using GCMS

The samples were extracted from crude *Angelica keiskei* using subcritical water. Using GC-MS analysis, the active compound, 2,3-dihydro-3,5-dihydroxy-6-methyl-4H-pyran-4-one (DDMP), was identified from a crude sample of *Angelica keiskei* to proceed with further experiments.

### 3.2. Cytotoxic Effect and Morphological Alteration of Angelica keiskei on HepG2 Cells

**Figure 1:**
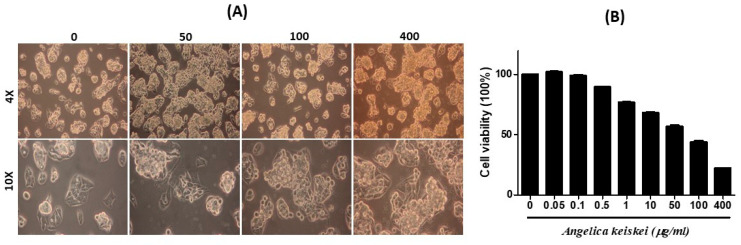
The MTT assay reveals both physical and physiological alterations, with the IC50 observed within the 50 to 100 µg/mL range. Data represent the mean ± SD from three independent experiments. *p* < 0.05 was considered statistically significant. (**A**) Microscopy images of HepG2 cells treated with *Angelica keiskei* (0, 50, 100, and 400 µg/mL) Increasing extract concentrations causes changes in cell morphology and reduced confluency. (**B**) Cell viability of HepG2 cells treated with *Angelica keiskei* at different concentrations (0 to 400 µg/mL) was assessed using MTT assay. Cell viability decreases in a dose-dependent manner.

The cytotoxic effect of various doses of *Angelica keiskei* was assessed in HepG2 cells using an MTT assay, which revealed reduced cell viability in a dose-dependent manner. At a concentration of 100 µg/mL, *Angelica keiskei* hindered the growth of HepG2 cells, as indicated by the IC50 value. Preliminary findings on the effects of *Angelica keiskei* on HepG2 cells were evaluated using the MTT assay (Figure 1). Morphological changes were noted in HepG2 cells following *Angelica keiskei* treatment in a dose-dependent manner.

### 3.3. Protein Expression Alteration Using the Apoptotic Array Analysis

**Figure 2:**
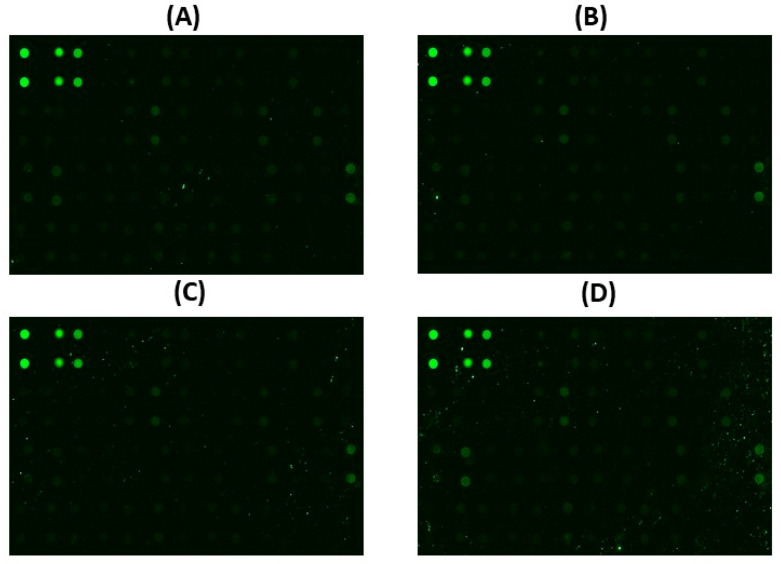
Antibody array detection of apoptotic proteins in treated HepG2 cells. Fluorescence images show apoptotic profiling using an immunogen array. Conditions: (**A**) Control; (**B**) 50 µg/mL; (**C**) 100 µg/mL; (**D**) 400 µg/mL.

By employing advanced antibody array technology with meticulous precision, we conducted a thorough examination to discern intricate variations in apoptotic proteins within the cellular milieu of HepG2 cells. This study focuses on the effect of *Angelica keiskei* treatment across a spectrum of concentrations, specifically at four distinct levels. This in-depth analysis encompassed the scrutiny of a comprehensive panel comprising a total of 43 proteins mentioned in Table 2, to unravel the specific molecular responses induced by treatment. In the illustrative data presented in Figure 2, the differential expression patterns of selected proteins, namely IGF-II, IGFBP-1, STNF-R1, DR6, Fas, TRAILR-3, TRAILR-4, HSP60, P21, BIM, HTRA, and SMAC, were closely observed. These proteins exhibited distinctive alterations in their expression profiles in direct response to *Angelica keiskei* treatment at dosages spanning 50, 100, and 400 µg/mL. This thorough examination of protein dynamics at varying concentrations provides a detailed understanding of how *Angelica keiskei* influences apoptotic proteins in HepG2 cells. Together, these findings provide a deeper insight into the complex modulation of apoptotic proteins induced by varying concentrations of *Angelica keiskei*. The detailed insights derived from this comprehensive analysis enhance our understanding of the specific impact of treatment on apoptotic pathways within HepG2 cells, thereby advancing our knowledge of targeted therapeutic interventions.

### 3.4. Identification of Differentially Expressed Apoptotic Proteins Using Cluster Heatmap

**Figure 3:**
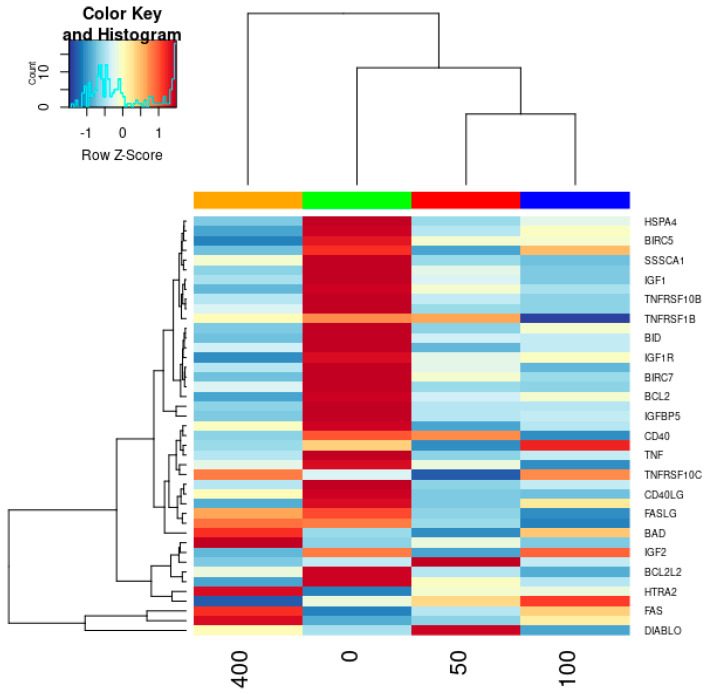
Cluster heat map of differentially expressed apoptotic proteins. Relative protein expression changes in HepG2 cells treated with increasing concentrations of *Angelica keiskei*. Color gradient denotes log-fold changes ranging from −1 to +1.

The effects of *Angelica keiskei* treatment on HepG2 cells were further elucidated through a visual representation of a cluster heat map, as illustrated in Figure 3. This analytical tool visually represents the observed changes and offers clear insights into the treatment-induced response levels. Close examination of the cluster heat map revealed that *Angelica keiskei* treatment elicited a notable modulation in the relative abundance of specific proteins within HepG2 cells. Notably, BAD, HTRA2, and IGFBP1 exhibited a relative increase in abundance, suggesting the upregulation of their expression levels. In contrast, IGFBP5 and CDKN1A were downregulated in response to *Angelica keiskei* treatment. These differential expression patterns are particularly significant because these proteins are integral components of apoptotic signaling pathways and play pivotal roles in cellular responses, particularly those related to programmed cell death. The observed alterations in protein expression, as revealed by the cluster heat map, indicated potential co-regulatory mechanisms implicated in the induction of HCC cell death following *Angelica keiske*i treatment. It is noteworthy that these modulation patterns are not uniform but rather exhibit dosage-dependent trends, emphasizing the nuanced and context-specific nature of the treatment response. The intricate interplay of these proteins in apoptotic pathways, as highlighted by their differential expression patterns, further contributes to our understanding of the molecular mechanisms involved in *Angelica keiskei*-mediated responses in HepG2 cells. These comprehensive insights provide a foundation for a more intricate understanding of how treatments affect apoptotic signaling pathways, thereby enhancing our understanding of potential therapeutic implications for hepatocellular carcinoma.

### 3.5. Volcano Scatter Plot for Differentially Expressed Cell Death Proteins

**Figure 4:**
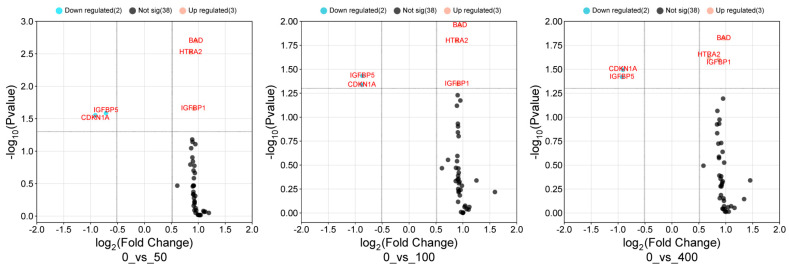
Volcano plot of apoptotic protein expression in response to treatment. Log-fold change representation of protein expression patterns for BAD, HTRA2, IGFBP1, IGFBP5, and CDKN1A. The graph illustrates the relative changes in protein expression levels between treated and untreated conditions. Each protein is individually depicted, providing a visual comparison of their respective log-fold changes in response to the experimental conditions.

A volcano scatter plot, shown in Figure 4, comprehensively illustrates the complexities of the changes in protein expression in response to *Angelica keiskei* treatment. This graphical representation provides a detailed description of the log-fold changes in the expression patterns of key apoptotic proteins, namely BAD, HTRA2, IGFBP1, IGFBP5, and CDKN1A. Remarkably, all of these apoptotic proteins exhibited a consistent trend of upregulated expression across all treatment groups, as indicated by logarithmic *p*-values exceeding 1.3. The upregulated proteins are distinctly highlighted in red on the plot, offering a visual indication of their increased abundance in response to *Angelica keiskei* treatment. The proteins that did not exhibit statistical significance are indicated in blue. Within this context, IGFBP5 has emerged as particularly noteworthy, displaying a significantly higher log-fold change than the other proteins, approaching a value of 2.0. This elevated log-fold change underscores the substantial impact of *Angelica keiskei* treatment on the expression levels of IGFBP5. The decision to use (−log10 (*p*-value > 1.3)) as a highlighting criterion served to emphasize the most statistically significant proteins, providing a clear focus on those with a more pronounced response to treatment. The detailed information extracted from the volcano scatter plot enabled a nuanced interpretation of *Angelica keiskei*-induced alterations in apoptotic protein expression. The consistent upregulation of all studied proteins, along with the marked prominence of IGFBP5, offers valuable insight into the molecular dynamics underlying the treatment response. This level of detail is instrumental in unraveling the intricacies of the impact of *Angelica keiskei* on apoptotic signaling pathways, further enhancing our understanding of its potential therapeutic implications.

### 3.6. Protein/Protein Interaction Network Investigation

**Figure 5:**
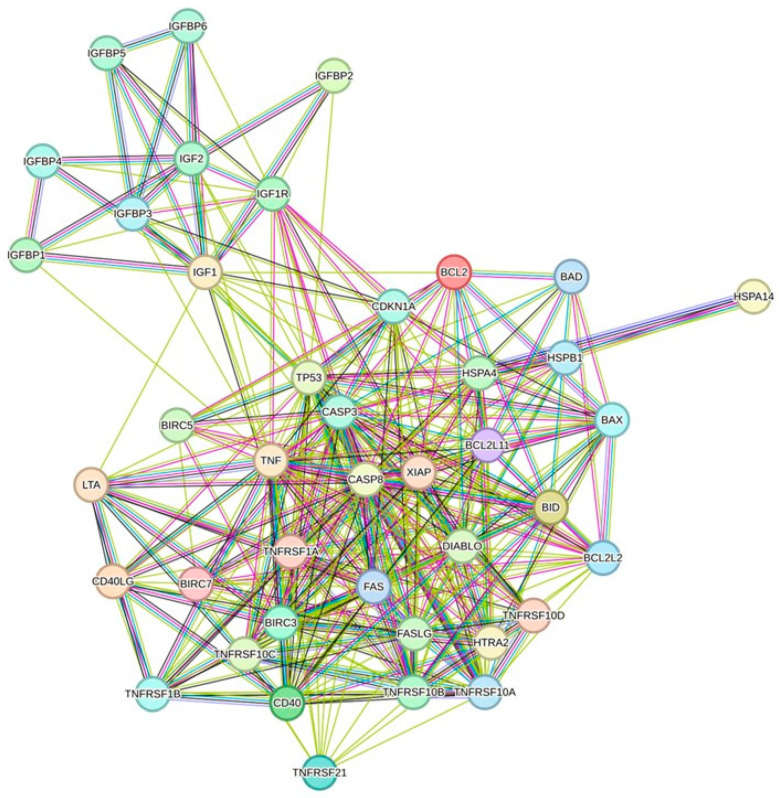
Protein/protein interaction network of selected apoptotic proteins. Network analysis highlighting the prediction of interacting protein partners during signaling. The nodes represent individual proteins, and the edges denote predicted interactions between them. This analysis provides insights into potential protein/protein interactions within signaling pathways, contributing to a comprehensive understanding of molecular networks associated with cellular signaling events. STRING analysis was used to predict functional relationships between BAD, HTRA2, IGFBP1, IGFBP5, and CDKN1A. The nodes represent proteins, while the edges represent predicted interactions. Edge colors indicate evidence sources: green for gene neighborhood, red for gene combination, blue for gene co-occurrence, purple for experimental, yellow for text mining, black for co-expression, and light blue for the curated database. The network focuses on key apoptotic regulators that are modulated by treatment.

The protein/protein interactions of BAD, HTRA2, IGFBP1, IGFBP5, and CDKN1A were analyzed using STRING analysis and are displayed in Figure 5. This analysis features the prediction of interacting protein partners during signaling, which helps annotate unknown functions. Our results showed that the expressed protein interactions, mainly those of the IGFBP family, are associated with the vital functions of tumor regulation behaviors, such as proliferation, migration, invasion, and adhesion, through different molecular mechanisms. Another interesting protein, BAD, plays a significant role in the regulation of apoptosis, and CDKN1A maintains DNA damage viability. However, HTRA2, a protein commonly referred to as Omi, is important for the apoptosis of liver cancer cells. It is mostly found in the mitochondria, where it is released into the cytoplasm during apoptosis. When released, HTRA2 promotes apoptosis by aiding caspase activation.

HTRA2’s capacity to bind to and block inhibitors of apoptosis proteins (IAPs), especially the X-linked inhibitor of apoptosis protein (XIAP), is the mechanism by which it induces apoptosis. By inhibiting caspase activity, XIAP typically stops apoptosis. HTRA2 eliminates this inhibition by binding to XIAP, which activates caspases and initiates apoptosis.

### 3.7. Principle Component Analysis

**Figure 6:**
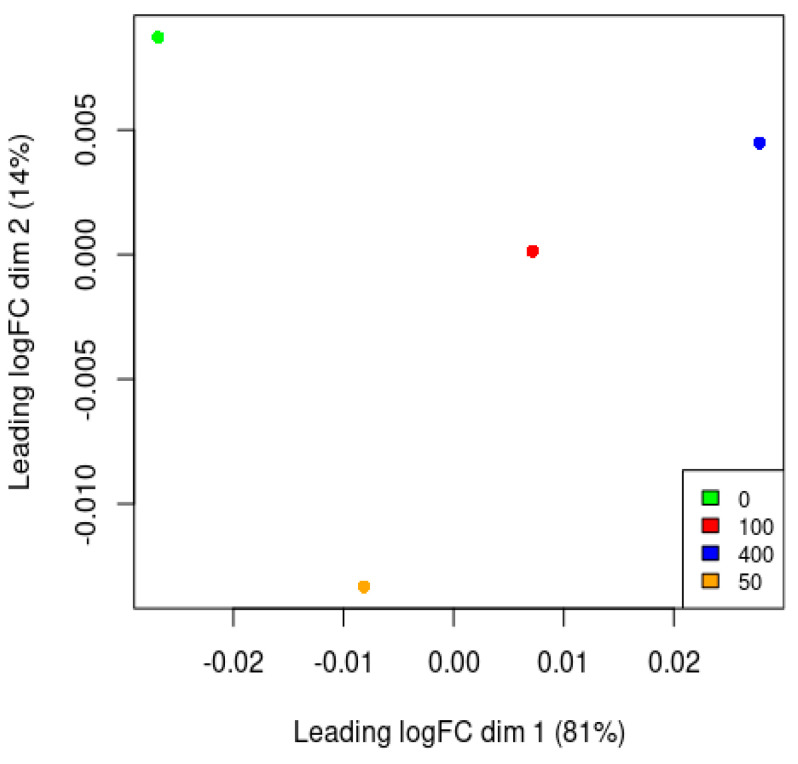
Principal component analysis (PCA) of apoptotic protein expression. PCA of apoptotic array data. PCA two-dimensional scatter plot represents the differential gene expression patterns of the treated and control group of samples. PC1: PCA Component 1 (81%), PC2: PCA Component 2 (14% variance).

The distribution of samples in the PC1–PC2 space is shown graphically in the scatter plot, which also reveals different clustering patterns between the treatment and control groups in Figure 6. This implies that there were discernible changes in the gene expression profiles of the two groups, which could be a sign of how the treatment affected apoptotic pathways. The significant variance explained by PC1 highlights the significance of this variable in identifying the main causes of variation in the data, which may represent the main distinctions between the treatment conditions. PC2 recorded extra variations that might be secondary patterns of variations in gene expression. Overall, PCA highlights the possible influence of experimental treatment on apoptotic pathways and offers insightful information on the differences in apoptosis-related gene expression patterns between the treated and control groups.

### 3.8. Voom: The Association Between the Mean and Variance

**Figure 7:**
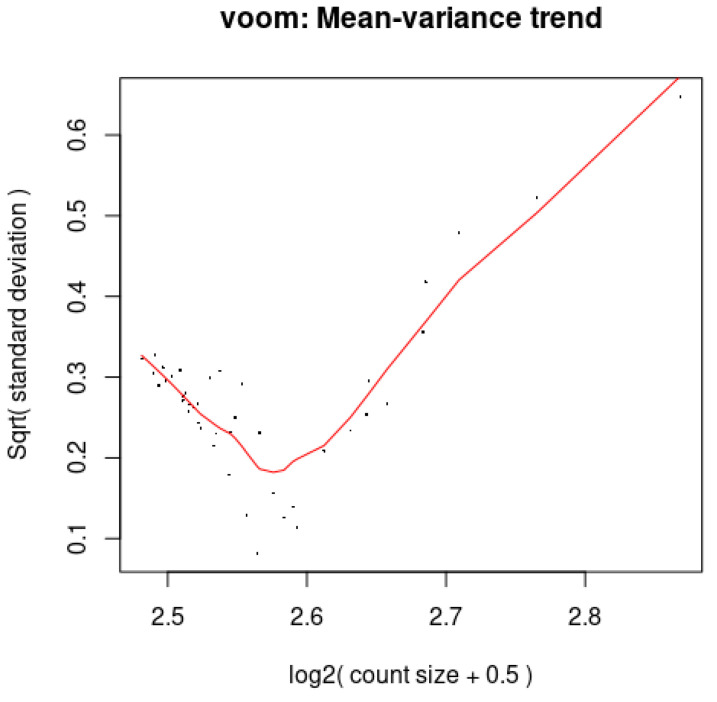
Voom plot depicting mean–variance trend of gene expression. The *x*-axis (corrected log2 expression values) ranges from 2.5 to 2.8, while the *y*-axis (expression variability) spans from 0.1 to 0.6. A red LOWESS curve fitted to the data shows a clear U-shaped pattern, indicating that moderately expressed genes exhibit the least variability, whereas genes with very low or high expression are more variable. The points show the mean-variance relationship in the data, which voom uses to calculate precision weights for linear modeling in RNA-seq or proteomics differential expression analysis.

A red LOWESS curve was plotted in Figure 7 to determine the trend in the dispersion of the dots. The trajectory of the curve has a distinctive ‘U-shape’, with less variance for genes with low expression levels, a minimum variance at intermediate expression levels, and a rise in variance for genes with high expression. Genes with very low or very high expression levels have more fluctuation in their expression measurements, according to this pattern, which suggests mean-dependent variability. Gene-wise means and variances of the microarray data are shown as black points with a LOWESS trend. Plots in the datasets were arranged according to increasing levels of biological variation.

### 3.9. Glimma Plots

**Figure 8:**
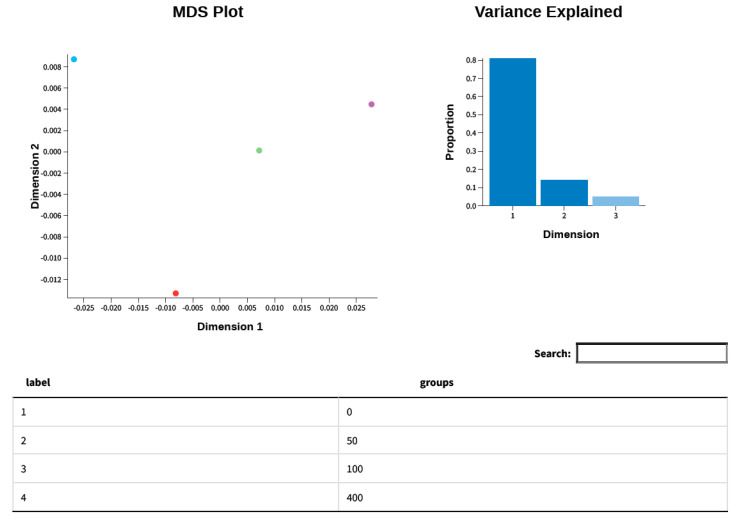
Multidimensional scaling (MDS) plot of gene expression data. The left side of the image displays the MDS plot, where the data points are plotted across the first two principal dimensions (Dimension 1 and Dimension 2). MDS is a means of visualizing the level of similarity of individual cases of a dataset. A bar plot that shows the percentage of variance explained by each MDS dimension is located on the right side. This is crucial in order to ascertain the extent to which the depicted dimensions account for the variability of the data, hence providing information regarding the quality of the data reduction. The height of the first bar in the bar plot indicates that the first-dimension accounts for the greatest percentage of variance. Each colored point represents a sample from a specific treatment group (0, 50, 100, and 400 µg/mL of *Angelica keiskei*).

Every dot in the MDS plot denotes an experimental sample in which the transcriptional output of many genes was measured, as represented in Figure 8. By reducing the dimensionality of the data using MDS, we can visually evaluate the similarities and differences in the transcription patterns of samples, with the first few dimensions representing the majority of the variation. Each dimension of the MDS plot explains a decreasing percentage of the overall variation, and samples that cluster together are more comparable than those that are far apart. This was employed in the exploratory analysis of an experiment to ascertain the factors that influenced sample variance and the strength of those effects.

### 3.10. Computational Analysis of 2,3-Dihydro-3,5-dihydroxy-6-methyl-4H-pyran-2-one and Insulin Growth Factor Binding Protein 1

**Figure 9:**
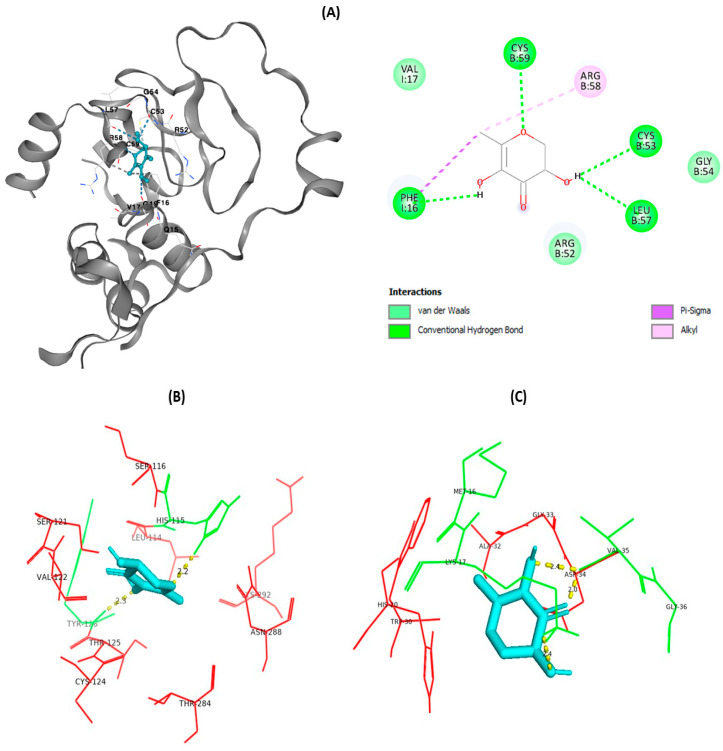
Molecular docking of DDMP with apoptotic proteins. (**A**) IGFBP1 (The position of amino acids in the protein are as follows R58, C59, R52, V17, G19, F16, Q15, (**B**) Bcl-2, and (**C**) p53 protein docking with DDMP has binding affinity −4.7, −5.3, and −5.4, respectively. Blue denotes the binding pocket; green and red denote the hydrophobic and hydrogen bonds, respectively, with respect to the consecutive amino acids. All docked images were visualized using the PyMOL software Version 2.4.0.

Initially, to evaluate the anticancer activity of *Angelica keiskei*, all compounds identified by GC-MS were docked to determine the binding affinity of each compound. Protein structures were sourced from the Protein Data Bank in PDB format, while the chemical structure of 2,3-Dihydro-3,5-dihydroxy-6-methyl-4H-pyran-2-one (DDMP) was obtained from the PubChem website. Docking was conducted within the binding pocket exhibiting the top-ranked score. In DDMP and IGFBP1, the protein interacted with the binding site through van der Waals interactions at the VAL17, GLY54, and ARG32 positions (Figure 9). Conventional hydrogen bond interactions were noted at the CYS53, CYS59, LEU57, and PHE16 positions, and alkyl interactions at the ARG58 position. An unfavorable acceptor/acceptor interaction was observed for LYS73. The compound’s binding affinity to the protein was −4.7 kcal/mol. Furthermore, DDMP was docked with p53 and bcl-2 protein; p53 and bcl-2 had a binding affinity of −5.4 and −5.3, respectively.

## 4. Discussion

Following surgical resection, certain tumors, such as breast, colon, and osteogenic sarcomas, and distant metastases to organs, including the lungs, liver, and bone, may develop quickly. Therefore, the development of novel anticancer drugs with antitumor and antimetastatic properties is necessary. The traditional uses of the roots of *Angelica keiskei* include galactagogic, analeptic, diuretic, and laxative effects. Although it has been said that *Angelica keiskei’*s roots and leaves help prevent cancer, hypertension, and coronary heart disease, the evidence for this is unclear [29]. In this study, we investigated the effects of different *Angelica keiskei* extract treatments on HepG2 cells. Normal untreated cells showed a regular morphology, but the treated cells showed granulation and clumping structures. Thus, *Angelica keiskei* extract has good anticancer activity against HepG2 cells. The MTT assay was performed to evaluate the desired concentration of *Angelica keiskei* extract in HepG2 cells. The cells were treated with concentrations of 0, 50, 100, and 400 µg/mL, and it showed that cell growth significantly decreased with increasing *Angelica keiskei* extract. A clinical study showed that regular drinkers with abnormal liver function tests showed considerably decreased levels of gamma-glutamyl transferase (GGT) after consuming *Angelica keiskei* extracts [30].

BAD belongs to the BCL-2 family and is recognized as a controlling factor of programmed cell death [31]. BAD is a pro-apoptotic protein that inhibits the pro-survival actions of these proteins when combined with BCL-xL and BCL-2 [32]. BAD’s pro-apoptotic activity is controlled by phosphorylation [33]. In human HCC, decreased p21WAF1 expression correlates with tumor progression and poor prognosis. p21WAF1 regulates programmed cell death through AKT [34], MAP kinase, and calcium phosphatase pathways. The Insulin-like Growth Factor Binding Protein 1 (IGFBP1) gene is associated with ovarian disease and HCC and influences gene expression and cellular responses to stimuli [35]. IGFBP1 plays a vital role in pathways involving hypoxia-inducible factor-dependent stem cell differentiation, and its upregulation negatively affects lymphangiogenesis in HCC [36]. Contradictory findings regarding blood levels and expression of IGFBP1 in patients with HCC have been published. According to a previous study, IGFBP1 may decrease tumor growth by blunting the IGF axis. In line with this, these findings suggested that IGFBP1, which functions as a tumor suppressor, would be a possible target for treating HCC [37,38,39]. It has been suggested that IGFBP5 in the blood of patients with NAFLD may signal the development of nonalcoholic steatohepatitis (NASH), a more severe liver disease, from NAFLD [40]. Umemura et al. identified the expression of IGFBP5 in HCC, suggesting that it may play an oncogenic role during the early stages of human hepatocarcinogenesis. They also suggested that IGFBP5 notably decreased the number of human HCC cells [41]. The heatmap illustrates the variation in gene expression related to the cell cycle. In HCC, CDKN1A and CDK4 were downregulated and upregulated, respectively. Mechanistic studies have revealed that the small nucleolar RNA host gene regulates CDKN1A expression, affecting HCC growth, along with other proteins [42]. By testing CDKN1A’s function as a regulator of proliferation, invasion, and responsiveness to anticancer therapies, one study demonstrated the inhibitory action of the gene [43]. HtrA2 expression is crucial for apoptosis in HCC cells, and the links between HtrA2 and HCC are narrow. A multidimensional analysis was used to determine the functional enrichment of HtrA2-related genes in HCC and to examine the association between HtrA2 and tumor immunity [44]. The relationship between HtrA2 and miR-519E may be significant because HtrA2 regulates tumor cell apoptosis. This finding suggests the need for further exploration of these connections in patients with HCC. The study identified a correlation between HtrA2 dysregulation and several transcription factors (IPF1, HFH4, PAX4, E4BP4, and ETF) known to regulate proliferation and mortality in various human cell types [45,46,47,48,49,50].

In our study, IGFBP5 and CDKN1A proteins were downregulated at higher doses in HCC cells, potentially leading to apoptosis. This was confirmed by cluster heatmap and volcano scatter plot analyses, which showed altered expression of key apoptosis-related proteins. As per ‘A Textbook of Liver Disease, 2018’ [51], AKT phosphorylates numerous proteins, such as the pro-apoptotic protein BAD, leading to its inactivation. Moreover, a substantial proportion of HCC tumors demonstrate reduced expression of the tumor suppressor gene products, phosphatase, and tensin homolog, which commonly suppress PI3K activity. Bad displaces Bax when it is not phosphorylated via preferential dimerization with Bcl-xL and Bcl-2. The liver mainly produces IGFBP-1 and can negatively regulate the activation of IGF-R, as it is known that the IGF signaling pathway plays an important role in the development and progression of HCC of IGF-R, as it is known that the IGF signaling pathway plays an important role in the development and progression of HCC. Studies on HepG2 cells have demonstrated that the MAPK pathway is the mechanism by which IL-1 mediates the induction of IGFBP-1 [52]. After AKE treatment, the elevated expression of IGFBP1 in HepG2 cells suggests that *IGFBP1* may be a transcriptional target of p53 in hepatic cells. One of the most frequently altered genes in HCC is the tumor suppressor p53. This transcription factor regulates several downstream target genes, including those involved in cell cycle progression, apoptosis, DNA repair, senescence, and metabolism [53]. Using protein microarray analysis, several proteins were upregulated by AKE. Molecular docking confirmed that IGFBP1 was upregulated in HepG2 cells. IGFBP1 binds to insulin growth factors (IGFs), modifying their effects and revealing that the Bcl-2 group of proteins Bad and Bid, including Bcl-2, may induce apoptosis regulated by p53. In many cell systems, the pro-apoptotic activity of the Bcl-2 family protein Bid is essential for death receptor-mediated apoptosis. The activity of Bid involves the relocation of its truncated form, tBid, to the mitochondria, facilitating the release of apoptogenic proteins such as cytochrome c, thus triggering apoptosis. Molecular docking confirmed the upregulation of one of these proteins in the HepG2 cells. IGFBP1 binds to insulin growth factors (IGFs), alters their effects, and is primarily produced in the liver. Previous studies have suggested that IGFBP1 may act as a tumor suppressor, although its exact mechanism remains unclear. Our ongoing research aims to delve deeper into the molecular mechanisms underlying *Angelica keiskei* extract.

A previous study has shown that DDMP triggers DNA strand breakage in a dose- and time-dependent manner [54]. Another study showed that the cellular response to generated DNA adducts is not well understood. Researchers used a variety of SULT1A1-competent cell models comprising primary hepatocytes exposed to the primary phase I metabolite 1′-hydroxymethyleugenol (OH-ME).

DNA damage primarily triggers the ATR-mediated DNA damage response, as evidenced by the phosphorylation of CHK1 and histone 2AX, followed by p53 accumulation and phosphorylation of CHK2. Consistent with these findings, DNA adducts slow replication and cause replication forks to halt. OH-ME treatment decreased cell viability, notably in cell lines containing wild-type p53, and caused apoptotic cell death, which was prevented by pan-caspase inhibition. Further research has established that mitochondrial apoptosis is an important route of cell death. ME-induced DNA damage increases the expression of the p53-responsive genes NOXA and PUMA, activates Bax, and releases cytochrome c, followed by the cleavage of caspase-9 and caspase-3. Finally, we revealed that p53 is critical for OH-ME-induced cell death, as indicated by the decreased pro-apoptotic gene expression, dramatically diminished Bax activation, and inhibition of cell death following genetic or pharmacological p53 suppression. Our findings show for the first time that ME-induced DNA damage promotes replication stress and mitochondrial death via the p53-Bax pathway [55]. SULTs are abundant in metabolically active or hormonally sensitive tissues such as the liver and numerous extrahepatic tissues. SULT expression is regulated by its isoforms, tissues, sex, and developmental factors [56]. When ionizing radiation (I.R.) causes double-strand DNA breaks (DSBs), mitotically dividing germ cells stop dividing by activating conserved DNA damage checkpoints [57].

p53’s pro-apoptotic function remains consistent throughout evolution, primarily through the transcriptional induction of BH3-only domain proteins. However, the specific target genes through which p53 halts the cell cycle vary between studies. p53 contributes to genome preservation by providing time for DNA repair mechanisms to address lesions before cell growth resumes. In response to DNA damage preceding the S phase, p53 arrests the cell cycle in the G1 phase, partly by inducing the cyclin-dependent kinase inhibitor CDKN1A, also known as p21 [58]. In addition to its well-established function in controlling the cell cycle in response to DNA damage, p53 has also been directly linked to the control and involvement in other DNA repair processes. According to the National Library of Medicine, the HTRA2-A serine protease is encoded by this gene. This protein is located in the endoplasmic reticulum and interacts with the mitogen-activated protein kinase 14 in an alternatively spliced form. Additionally, the protein is found in the mitochondria, where it is released into the cytosol in response to apoptotic stimulation. This protein is assumed to cause apoptosis by binding to the apoptosis-inhibitory protein baculoviral IAP repeat-containing 4. This protein has also been shown to localize in the nucleus. This gene transcribes various isoforms into several variants via alternative splicing.

## 5. Conclusions

In conclusion, our work shows that *Angelica keiskei* extract has favorable impacts on cell survival and apoptotic markers in HepG2 cells, implying possible anticancer effects. By using in silico molecular docking and proteomic analysis, we investigated possible molecular interactions and discovered binding affinities to apoptotic proteins, such as p53, that could contribute to the extract’s effects. We recognize, therefore, that these in silico results are preliminary and need more experimental validation to confirm the identified binding relationships and particular apoptotic pathways. To confirm these in silico findings effectively and clarify the precise mechanisms of action, future research will be needed. This will include evaluations of important proteins such as IGFBP1, IGFBP5, BAD, and Bcl-2. Further in vitro and in vivo models will enable us to better comprehend the extract’s therapeutic potential and examine its impact on hepatocellular carcinoma (HCC). These findings are preliminary but encouraging, and more investigation is required to validate the extract’s application as an anticancer approach in HCC. It is important to note that these findings are preliminary and derived from in vitro experiments; thus, further in vivo studies are necessary to validate the observed effects and elucidate the underlying mechanisms.

## Figures and Tables

**Table 1:** Subcritical water extraction conditions for the recovery of bioactive compounds from *Angelica keiskei.* The following acronyms correspond to extraction conditions shown in the table, and No means no ethanol was added; water was used as the sole solvent. Each treatment was conducted in a batch reactor under varying temperatures (140–170 °C), pressures (maintained at 3 MPa), reaction times (10 min), solid-to-liquid ratios (30 or 45 mg/mL), and solvent compositions (water or 50% ethanol). Acronyms denote specific conditions: ‘B’ indicates baseline treatments using water only, ‘E’ signifies the inclusion of ethanol, and numerical identifiers differentiate between treatment variations.

	Temperature (°C)	Pressure (MPa)	Reaction Time (min)	Solid/Liquid Ratio (mg/mL)	Ethanol	Acronyms
1	160	30	10	30	No	B1
2	160	30	10	45	No	B2
3	170	30	10	30	No	B3
4	140	30	10	30	No	B4
5	150	30	10	30	No	B5
6	160	30	10	30	50%	B-E-1

**Table 2:** List of the antibodies used to perform the human apoptotic array assay to analyze the apoptotic activity of the *AKE* against the HepG2 cells.

RayBio^®^ Human Apoptosis Antibody Array G1												
Detect 43 Apoptotic Markers in One Experiment												
**1**	**2**	**3**	**4**	**5**	**6**	**7**	**8**	**9**	**10**	**11**	**12**	**13**
Pos 1	Pos 2	Pos 3	Neg	Neg	bad	bax	bcl-2	bcl-w	BID	BIM	Casp 3	Casp 8
Pos 1	Pos 2	Pos 3	Neg	Neg	bad	bax	bcl-2	bcl-w	BID	BIM	Casp 3	Casp 8
CD40	CD40L	cIAP-2	cytoC	DR6	Fas	FasL	NEG	HSP27	HSP60	HSP70	HTRA	IGF-I
CD40	CD40L	cIAP-2	cytoC	DR6	Fas	FasL	NEG	HSP27	HSP60	HSP70	HTRA	IGF-I
IGF-II	IGFBP-1	IGFBP-2	IGFBP-3	IGFBP4	IGFBP-5	IGFBP-6	IGF-1sR	Iivin	p21	p27	p53	SMAC
IGF-II	IGFBP-1	IGFBP-2	IGFBP-3	IGFBP-4	IGFBP-5	IGFBP-6	IGF-1sR	livin	p21	p27	p53	SMAC
Survivin	sTNF-R1	sTNF-R2	TNF-alpha	TNF-beta	TRAIL R1	TRAIL R-2	TRAIL R-3	TRAIL R-4	XIAP	NEG	NEG	NEG
Survivin	sTNF-R1	sTNF-R2	TNF-alpha	TNF-beta	TRAIL R1	TRAIL R-2	TRAIL R-3	TRAIL R-4	XIAP	NEG	NEG	NEG

## Data Availability

Data is contained within the article.

## Supplementary Materials

The following supporting information can be downloaded at: https://www.mdpi.com/article/10.3390/cimb47060401/s1.

## Abbreviations

The following abbreviations are used in this manuscript:

A.K.	*Angelica keiskei*
BAD	BCL2 antagonist of cell death
CDKN1A	Cyclin-dependent kinase inhibitor 1A
DDMP	2,3-Dihydro-3,5-dihydroxy-6-methyl-4H-pyran-4-one
GC-MS	Gas chromatography-mass spectrometry
HCC	Hepatocellular carcinoma
HepG2	Hepatoblastoma cell line
HTRA2	HtrA Serine Peptidases 2
IGFBP1	Insulin-like growth factor binding proteins1
IGFBP5	Insulin-like growth factor binding proteins5
MEM	Minimum Essential Medium
MTT	3-(4,5-dimethylthiazol-2-yl)-2,5-diphenyl tetrazolium bromide
