# Supplementary material for: Pharmacological Evaluation of Angelica keiskei Extract: Molecular Interaction Analysis in Hepatocellular Carcinoma
Source: Curr Issues Mol Biol. 2025 May 29;47(6):401. doi: 10.3390/cimb47060401 (PMC12191638; doi:10.3390/cimb47060401)

Sample Information

Sample Name = Sample_M
C:\GCMSsolution\Data\Project1\DATA\2021\1103\Sample_M.qgd

Library Search Report

<< Target >>
Line#:1 R.Time:5.710(Scan#:143) MassPeaks:303
RawMode:Averaged 5.705-5.715(142-144) BasePeak:43.00(53156)
BG Mode:Calc. from Peak Group 1 - Event 1

Target Spectrum

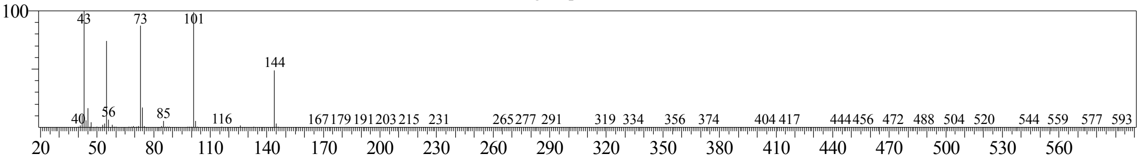

Hit#:1 Entry:12703 Library:NIST11.lib
SI:92 Formula:C6H8O4 CAS:10230-62-3 MolWeight:144 RetIndex:1173
CompName:2,4-Dihydroxy-2,5-dimethyl-3(2H)-furan-3-one $$ 2,4-Dihydroxy-2,5-dimethyl-3(2H)-furanone $$ 2,5-Dimethyl-2,4-dihydroxy-3(2H)-furanone $$

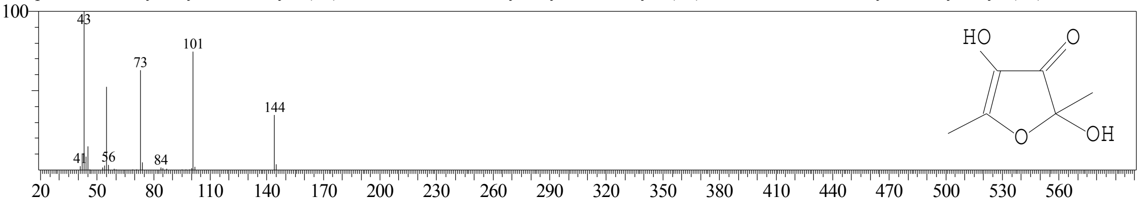

Hit#:2 Entry:42591 Library:Wiley9.lib
SI:81 Formula:C6H8O4 CAS:28564-83-2 MolWeight:144 RetIndex:0
CompName:2,3-Dihydro-3,5-dihydroxy-6-methyl-4H-pyran-4-one

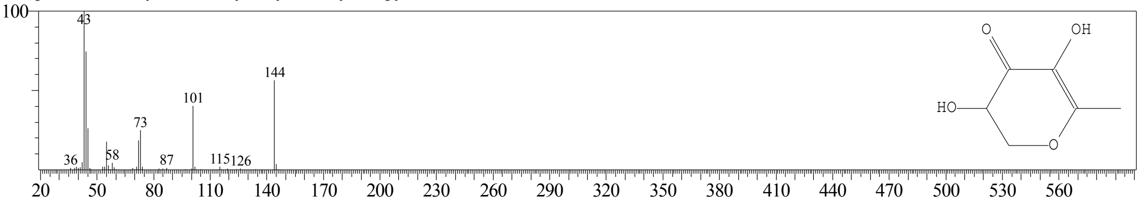

Hit#:3 Entry:170405 Library:Wiley9.lib
SI:80 Formula:C13H24O2 CAS:0-00-0 MolWeight:212 RetIndex:0
CompName:(E) Ethyl Undec-2-enoate

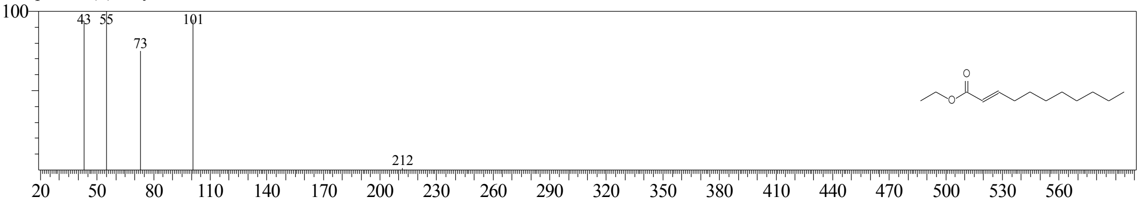

Hit#:4 Entry:42586 Library:Wiley9.lib
SI:79 Formula:C6H8O4 CAS:28564-83-2 MolWeight:144 RetIndex:0
CompName:4H-Pyran-4-one, 2,3-dihydro-3,5-dihydroxy-6-methyl- (CAS) $$ 3,5-DIHYDROXY-2-METHYL-5,6-DIHYDROPYRAN-4-ONE $$ 4H-PYRAN-4

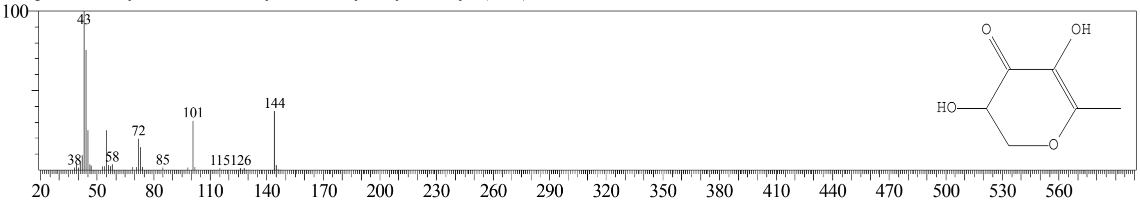

Hit#:5 Entry:42587 Library:Wiley9.lib
SI:79 Formula:C6H8O4 CAS:28564-83-2 MolWeight:144 RetIndex:0
CompName:4H-Pyran-4-one, 2,3-dihydro-3,5-dihydroxy-6-methyl- (CAS) $$ 3,5-DIHYDROXY-2-METHYL-5,6-DIHYDROPYRAN-4-ONE $$ 4H-PYRAN-4

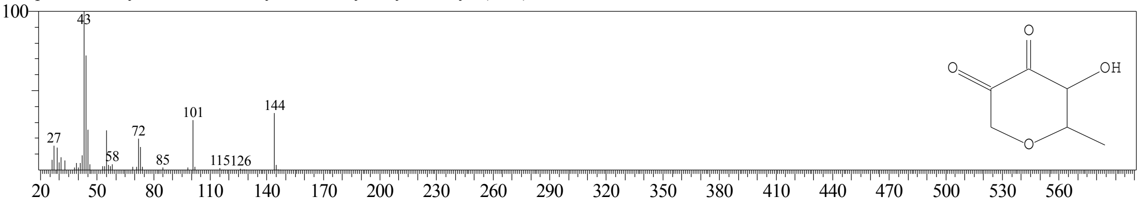

<< Target >>
Line#:2 R.Time:5.860(Scan#:173) MassPeaks:265
RawMode:Averaged 5.855-5.865(172-174) BasePeak:57.05(15281)
BG Mode:Calc. from Peak Group 1 - Event 1

Target Spectrum

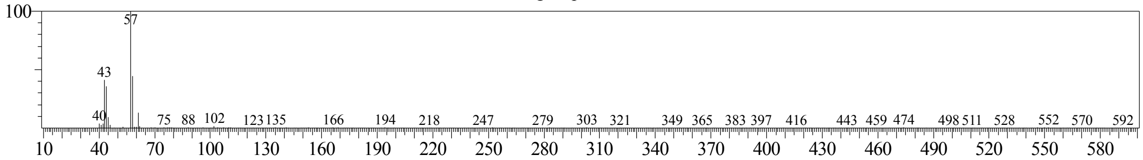

Hit#:1 Entry:2215 Library:NIST11.lib
SI:87 Formula:C4H6O3 CAS:19444-84-9 MolWeight:102 RetIndex:1013
CompName:2-Hydroxy-gamma-butyrolactone $$ 3-Hydroxydihydro-2(3H)-furanone # $$

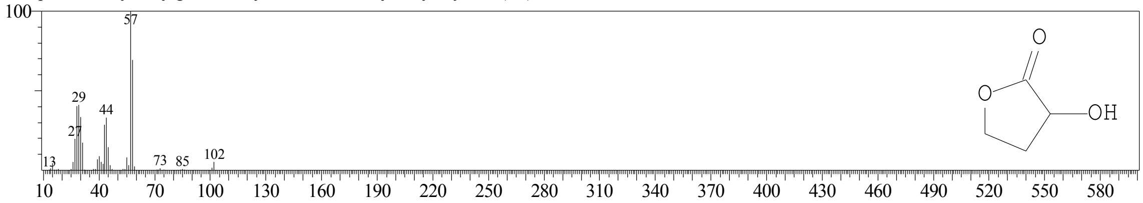

Hit#:2 Entry:4591 Library:Wiley9.lib
SI:85 Formula:C4H8O2 CAS:453-20-3 MolWeight:88 RetIndex:0
CompName:3-Furanol, tetrahydro- (CAS) $$ 3-Hydroxytetrahydrofuran $$ TETRAHYDRO-3-HYDROXY-FURAN $$ Tetrahydro-3-furanol $$ TETRAHYDR(

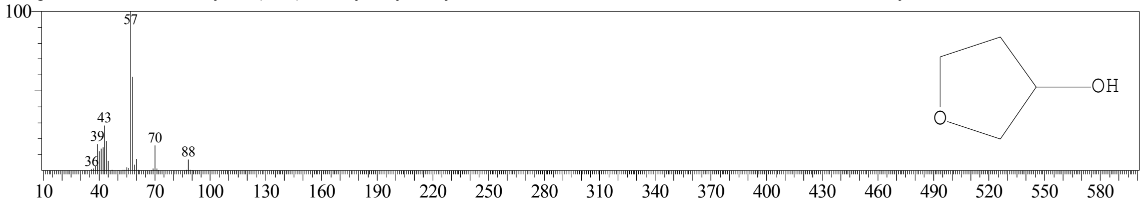

Hit#:3 Entry:2295 Library:Wiley9.lib
SI:84 Formula:C3H8O2 CAS:504-63-2 MolWeight:76 RetIndex:0
CompName:1,3-Propanediol (CAS) $$ PG $$ 2-Deoxyglycerol $$ Propane-1,3-diol $$ Trimethylene glycol $$ 1,3-Dihydroxypropane $$ 1,3-Propylene glycol $

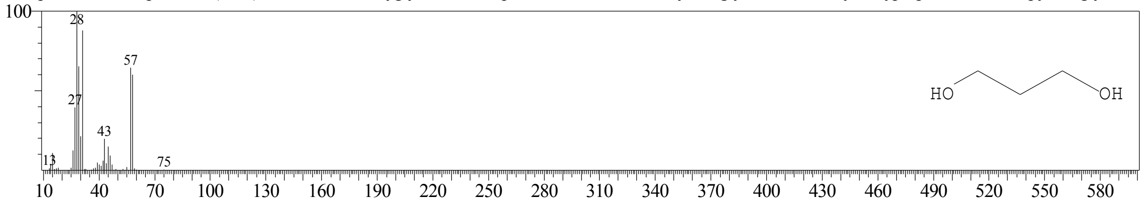

Hit#:4 Entry:4593 Library:Wiley9.lib
SI:83 Formula:C4H8O2 CAS:453-20-3 MolWeight:88 RetIndex:0
CompName:3-Furanol, tetrahydro- (CAS) $$ 3-Hydroxytetrahydrofuran $$ TETRAHYDRO-3-HYDROXY-FURAN $$ Tetrahydro-3-furanol $$ TETRAHYDR(

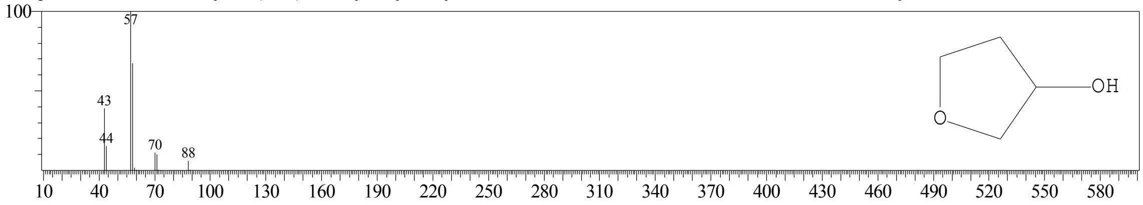

Hit#:5 Entry:992 Library:NIST11.lib
SI:83 Formula:C4H8O2 CAS:86087-23-2 MolWeight:88 RetIndex:777
CompName:(S)-(+)-3-Hydroxytetrahydrofuran $$ Tetrahydro-3-furanol # $$

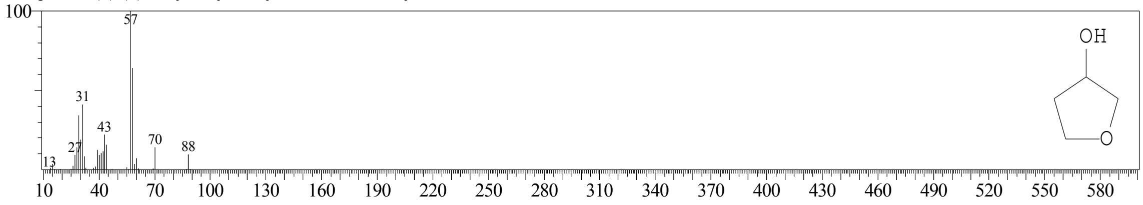

<< Target >>
Line#:3 R.Time:6.690(Scan#:339) MassPeaks:297
RawMode:Averaged 6.685-6.695(338-340) BasePeak:43.00(89516)
BG Mode:Calc. from Peak Group 1 - Event 1

Target Spectrum

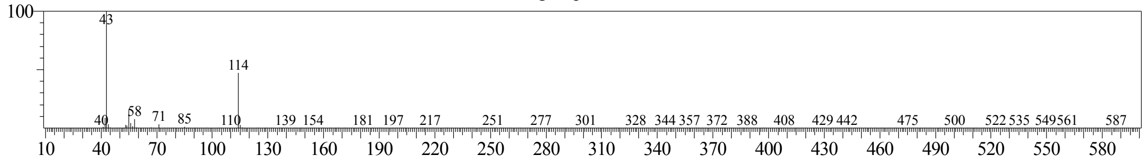

Hit#:1 Entry:3930 Library:NIST11.lib
SI:87 Formula:C5H6O3 CAS:37830-90-3 MolWeight:114 RetIndex:975
CompName:1,3-Dioxol-2-one,4,5-dimethyl- $$ 4,5-Dimethyl-1,3-dioxol-2-one # $$

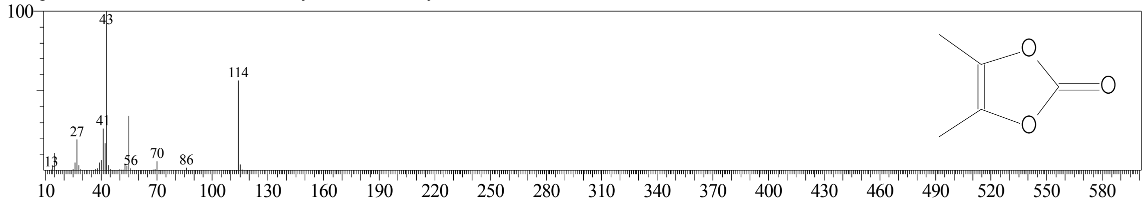

Hit#:2 Entry:14691 Library:Wiley9.lib
SI:86 Formula:C5H6O3 CAS:0-00-0 MolWeight:114 RetIndex:0
CompName:3 - methyl - tetrahydrofuran - 2,4 - di - one

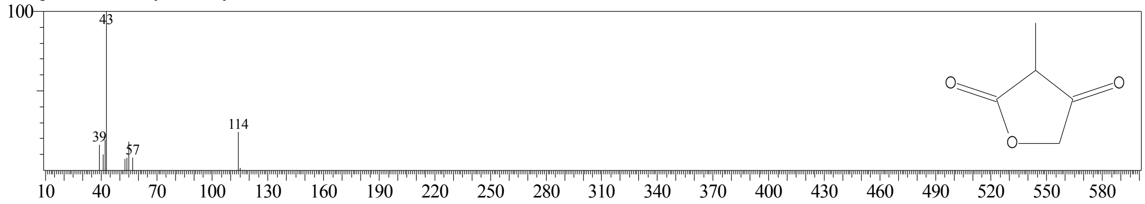

Hit#:3 Entry:15217 Library:Wiley9.lib
SI:84 Formula:C6H14N2 CAS:106-58-1 MolWeight:114 RetIndex:0
CompName:N,N'-Dimethylpiperazine $$ Piperazine, 1,4-dimethyl- $$ Lupetazine $$ 1,4-Dimethylpiperazine $$ PIPERAZIN, 1,4-DIMETHYL- $$ N,N-Dimeth

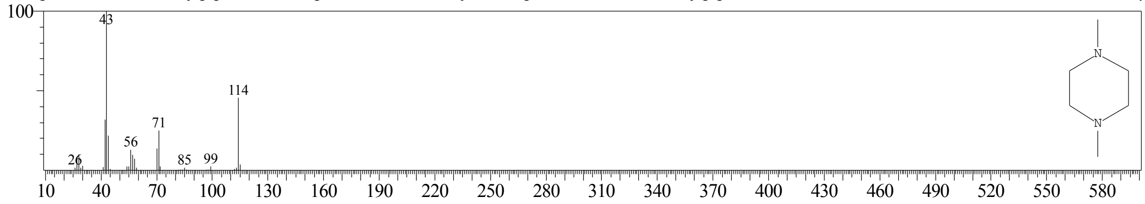

Hit#:4 Entry:14989 Library:Wiley9.lib
SI:84 Formula:C6H10O2 CAS:25465-18-3 MolWeight:114 RetIndex:0
CompName:1,4-Dioxin, 2,3-dihydro-5,6-dimethyl- (CAS) $$ 5,6-DIMETHYL-2,3-DIHYDRO-1,4-DIOXIN $$ p-Dioxin, 2,3-dihydro-5,6-dimethyl- (CAS) $$ 2

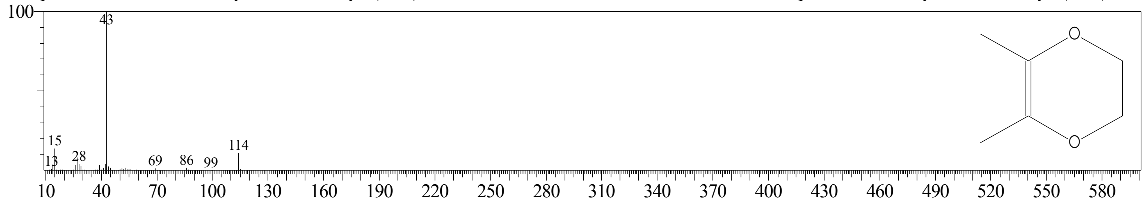

Hit#:5 Entry:3971 Library:NIST11.lib
SI:84 Formula:C6H10O2 CAS:25465-18-3 MolWeight:114 RetIndex:859
CompName:1,4-Dioxin, 2,3-dihydro-5,6-dimethyl- $$ p-Dioxin, 2,3-dihydro-5,6-dimethyl- $$ 2,3-Dimethyl-1,4-dioxene $$ 5,6-Dimethyl-2,3-dihydro-1,4-dioxi

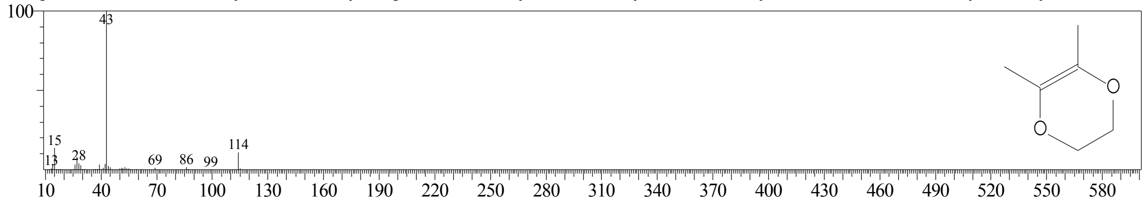

<< Target >>
Line#:4 R.Time:6.965(Scan#:394) MassPeaks:277
RawMode:Averaged 6.960-6.970(393-395) BasePeak:43.00(55456)
BG Mode:Calc. from Peak Group 1 - Event 1

Target Spectrum

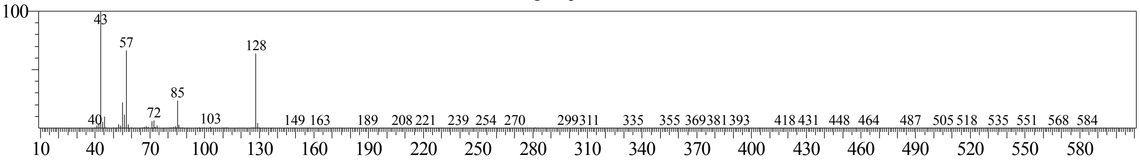

Hit#:1 Entry:25068 Library:Wiley9.lib
SI:95 Formula:C6H8O3 CAS:3658-77-3 MolWeight:128 RetIndex:0
CompName:2,5-Dimethyl-4-hydroxy-3(2H)-furanone $$ 3-FURANONE, 2,3-DIHYDRO-4-HYDROXY-2,5-DIMETHYL- $$ Furaneol $$ 2,5-dimethyl-4-hydro

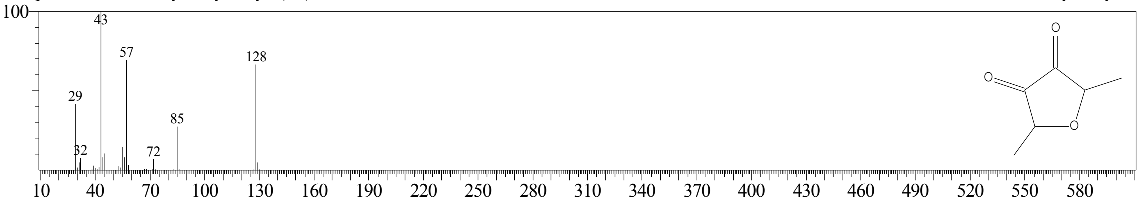

Hit#:2 Entry:25067 Library:Wiley9.lib
SI:95 Formula:C6H8O3 CAS:3658-77-3 MolWeight:128 RetIndex:0
CompName:2,5-Dimethyl-4-hydroxy-3(2H)-furanone $$ 3-FURANONE, 2,3-DIHYDRO-4-HYDROXY-2,5-DIMETHYL- $$ Furaneol $$ 2,5-dimethyl-4-hydro

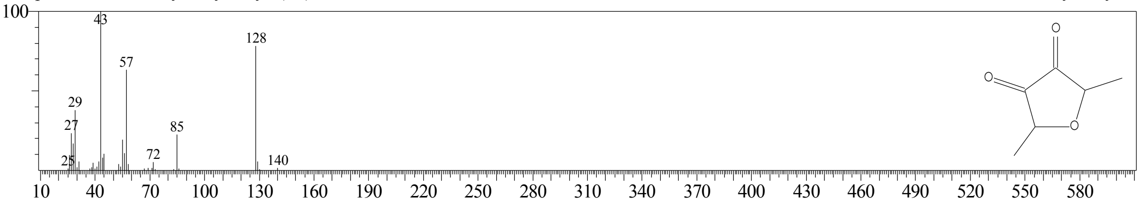

Hit#:3 Entry:25096 Library:Wiley9.lib
SI:94 Formula:C6H8O3 CAS:3658-77-3 MolWeight:128 RetIndex:0
CompName:HYDROXY DIMETHYL FURANONE $$ 4-HYDROXY-2,5-DIMETHYL-3(2H)-FURANONE $$ FURANEOL $$ FIRMENISH $$ 2,5-ANHYDR

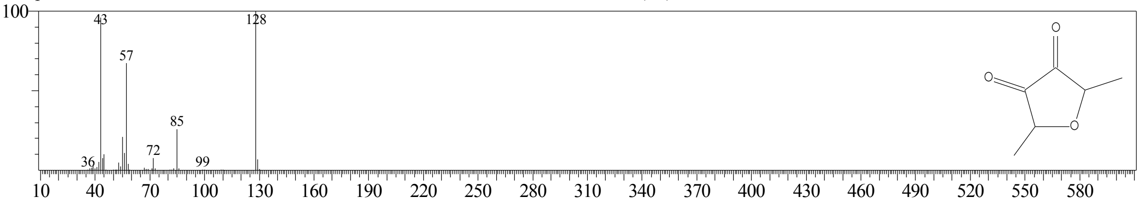

Hit#:4 Entry:7041 Library:NIST11.lib
SI:94 Formula:C6H8O3 CAS:3658-77-3 MolWeight:128 RetIndex:1022
CompName:2,5-Dimethyl-4-hydroxy-3(2H)-furanone $$ 3(2H)-Furanone, 4-hydroxy-2,5-dimethyl- $$ 3(2H)-Furanone, 2,5-dimethyl-4-hydroxy- $$ Alletone $$

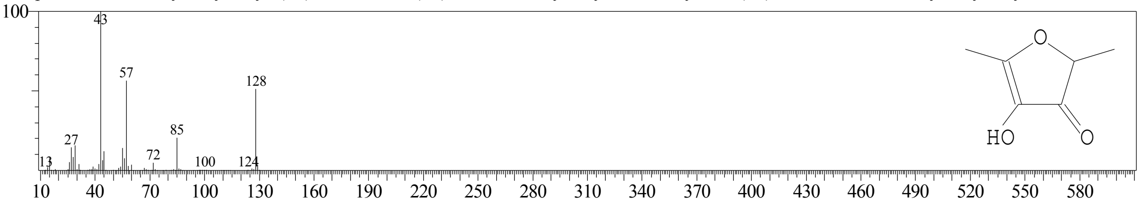

Hit#:5 Entry:25066 Library:Wiley9.lib
SI:94 Formula:C6H8O3 CAS:3658-77-3 MolWeight:128 RetIndex:0
CompName:2,5-Dimethyl-4-hydroxy-3(2H)-furanone $$ 3-FURANONE, 2,3-DIHYDRO-4-HYDROXY-2,5-DIMETHYL- $$ Furaneol $$ 2,5-dimethyl-4-hydro

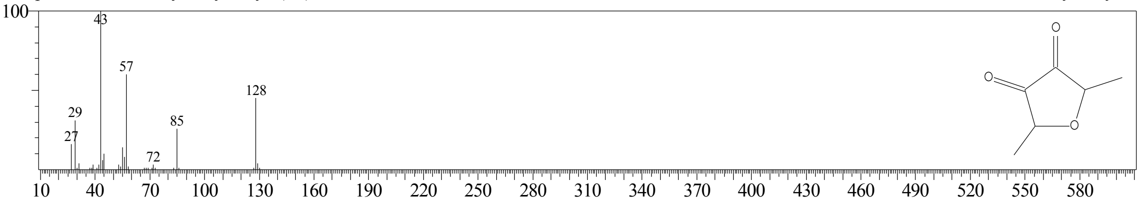

<< Target >>
Line#:6 R.Time:7.880(Scan#:577) MassPeaks:271
RawMode:Averaged 7.875-7.885(576-578) BasePeak:95.05(129884)
BG Mode:Calc. from Peak Group 1 - Event 1

Target Spectrum

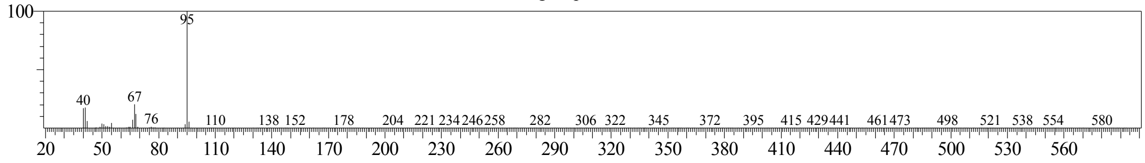

Hit#:1 Entry:1318 Library:NIST11.lib
SI:93 Formula:C5H5NO CAS:109-00-2 MolWeight:95 RetIndex:895
CompName:3-Pyridinol $$ 3-Hydroxypyridine $$ .beta.-Hydroxypyridine $$ 3-Oxopyridine $$ 3-Pyridol $$ 3-Pyridone $$

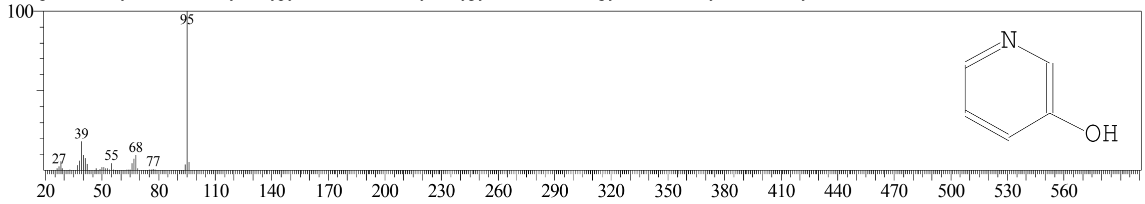

Hit#:2 Entry:5953 Library:Wiley9.lib
SI:93 Formula:C5H5NO CAS:109-00-2 MolWeight:95 RetIndex:0
CompName:3-Pyridinol (CAS) $$ 3-Hydroxypyridine $$ 3-Pyridol $$ 3-Pyridone $$ 3-Oxopyridine $$ .beta.-Hydroxypyridine $$ PYRIDIN-3-OL $$ SODIUM

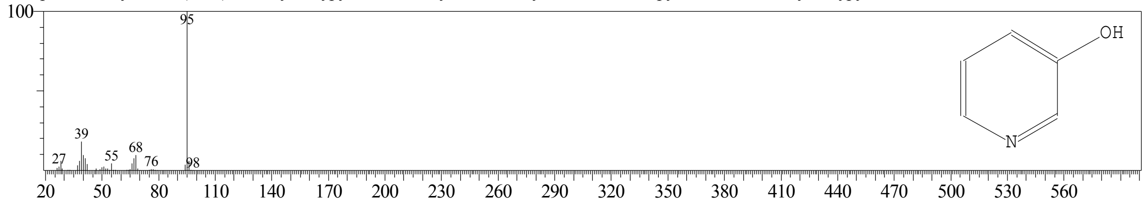

Hit#:3 Entry:5944 Library:Wiley9.lib
SI:92 Formula:C5H5NO CAS:142-08-5 MolWeight:95 RetIndex:0
CompName:2(1H)-Pyridinone (CAS) $$ 2-Pyridone $$ 2-Hydroxypyridine $$ 2-Pyridinol $$ 2-Pyridinone $$ 2-Oxopyridine $$ 2(1H)-Pyridone $$ .alpha.-Pyri

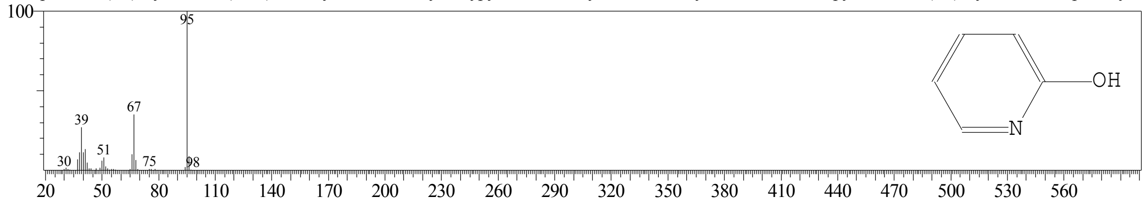

Hit#:4 Entry:5960 Library:Wiley9.lib
SI:92 Formula:C5H5NO CAS:626-64-2 MolWeight:95 RetIndex:0
CompName:4-Hydroxypyridine $$ 4-Pyridinol (CAS) $$ .gamma.-Hydroxypyridine $$ 4-Pyridone $$ 4-Pyridol $$ AI3-61970 $$ AIDS-081858 $$ BRN 010580

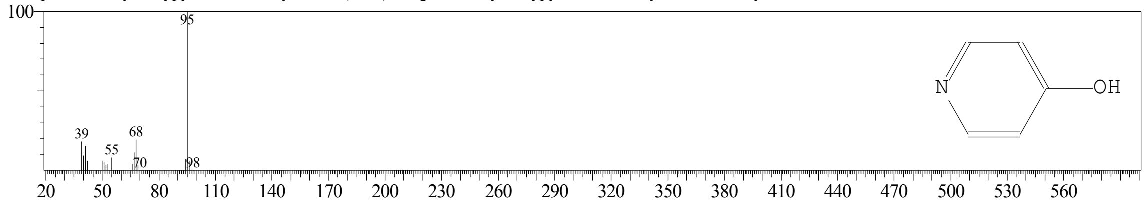

Hit#:5 Entry:5959 Library:Wiley9.lib
SI:92 Formula:C5H5NO CAS:626-64-2 MolWeight:95 RetIndex:0
CompName:4-Hydroxypyridine $$ 4-Pyridinol (CAS) $$ .gamma.-Hydroxypyridine $$ 4-Pyridone $$ 4-Pyridol $$ AI3-61970 $$ AIDS-081858 $$ BRN 010580

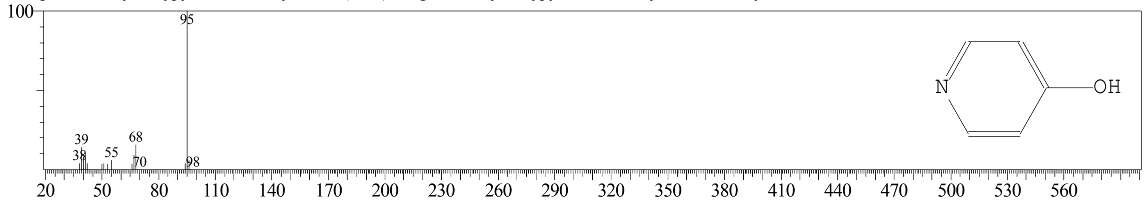

<< Target >>
Line#:7 R.Time:8.305(Scan#:662) MassPeaks:284
RawMode:Averaged 8.300-8.310(661-663) BasePeak:43.00(110224)
BG Mode:Calc. from Peak Group 1 - Event 1

Target Spectrum

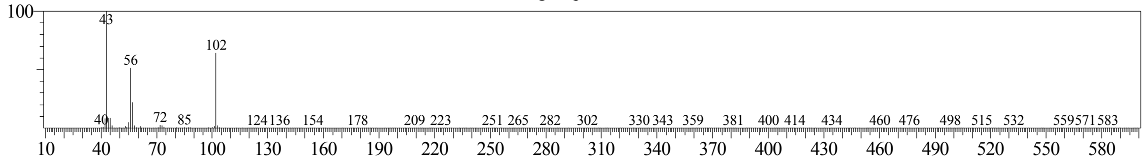

Hit#:1 Entry:42600 Library:Wiley9.lib
SI:86 Formula:C6H8O4 CAS:135366-64-2 MolWeight:144 RetIndex:0
CompName:2-acetyl-2-hydroxy-.gamma.-butyrolactone

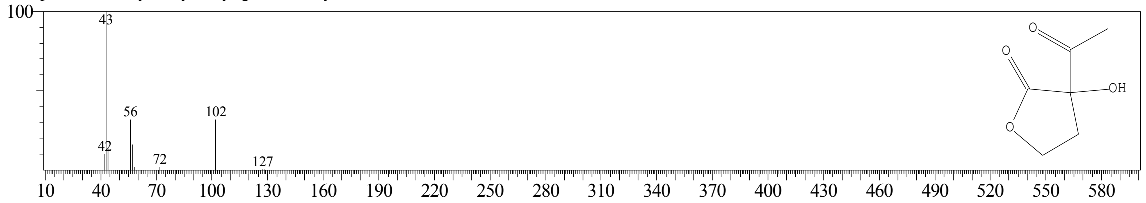

Hit#:2 Entry:16352 Library:Wiley9.lib
SI:83 Formula:C5H8O3 CAS:123-76-2 MolWeight:116 RetIndex:0
CompName:Pentanoic acid, 4-oxo- (CAS) $$ Levulinic acid $$ Levulic acid $$ Laevulinic acid $$ 4-Ketovaleric acid $$ 4-Oxopentanoic acid $$ 4-Oxovaleric a

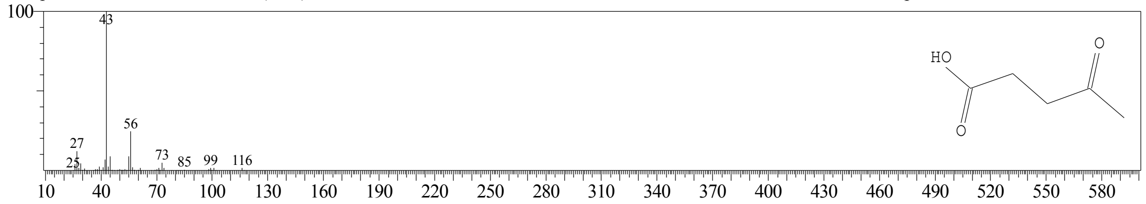

Hit#:3 Entry:16353 Library:Wiley9.lib
SI:83 Formula:C5H8O3 CAS:123-76-2 MolWeight:116 RetIndex:0
CompName:Pentanoic acid, 4-oxo- (CAS) $$ Levulinic acid $$ Levulic acid $$ Laevulinic acid $$ 4-Ketovaleric acid $$ 4-Oxopentanoic acid $$ 4-Oxovaleric a

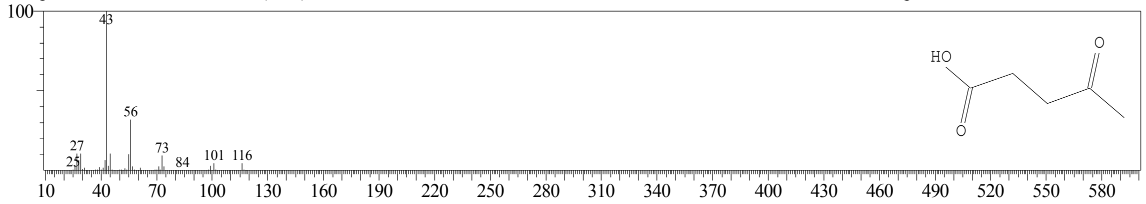

Hit#:4 Entry:16351 Library:Wiley9.lib
SI:83 Formula:C5H8O3 CAS:123-76-2 MolWeight:116 RetIndex:0
CompName:Pentanoic acid, 4-oxo- (CAS) $$ Levulinic acid $$ Levulic acid $$ Laevulinic acid $$ 4-Ketovaleric acid $$ 4-Oxopentanoic acid $$ 4-Oxovaleric a

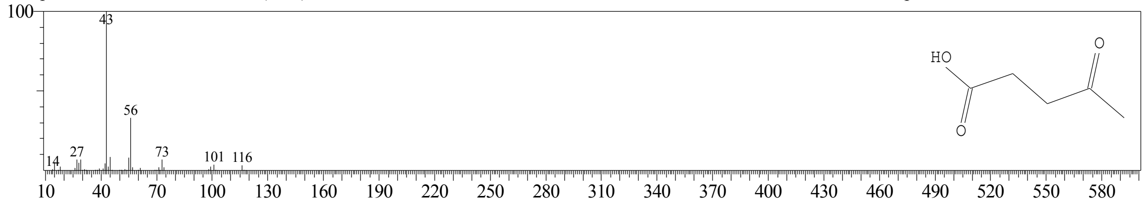

Hit#:5 Entry:2102 Library:NIST11.lib
SI:83 Formula:C4H7NO2 CAS:1192-20-7 MolWeight:101 RetIndex:1030
CompName:.alpha.-Amino-.gamma.-butyrolactone $$ 3-Aminodihydro-2(3H)-furanone $$ Homoserine lactone $$ 2(3H)-Furanone, 3-aminodihydro $$

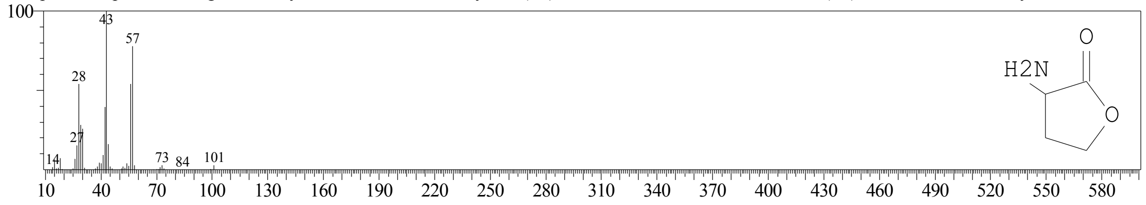

<< Target >>
Line#:8 R.Time:8.535(Scan#:708) MassPeaks:325
RawMode:Averaged 8.530-8.540(707-709) BasePeak:43.00(814735)
BG Mode:Calc. from Peak Group 1 - Event 1

Target Spectrum

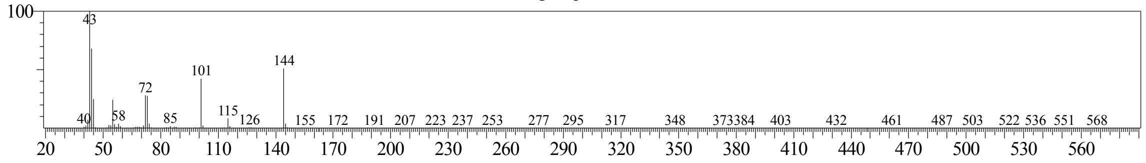

Hit#:1 Entry:42591 Library:Wiley9.lib
SI:96 Formula:C6H8O4 CAS:28564-83-2 MolWeight:144 RetIndex:0
CompName:2,3-Dihydro-3,5-dihydroxy-6-methyl-4H-pyran-4-one

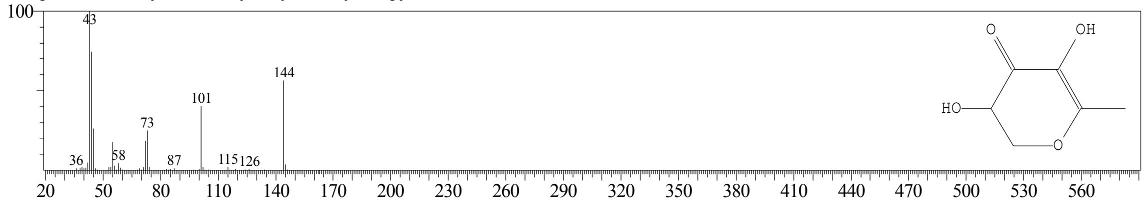

Hit#:2 Entry:42587 Library:Wiley9.lib
SI:94 Formula:C6H8O4 CAS:28564-83-2 MolWeight:144 RetIndex:0
CompName:4H-Pyran-4-one, 2,3-dihydro-3,5-dihydroxy-6-methyl- (CAS) $$ 3,5-DIHYDROXY-2-METHYL-5,6-DIHYDROPYRAN-4-ONE $$ 4H-PYRAN-4

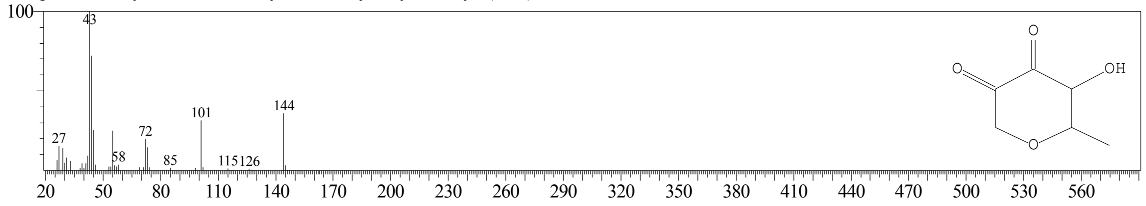

Hit#:3 Entry:12699 Library:NIST11.lib
SI:94 Formula:C6H8O4 CAS:28564-83-2 MolWeight:144 RetIndex:1269
CompName:4H-Pyran-4-one, 2,3-dihydro-3,5-dihydroxy-6-methyl- $$ 3,5-Dihydroxy-6-methyl-2,3-dihydro-4H-pyran-4-one $$ 2,3-dihydro-3,5-dihydroxy--6-m

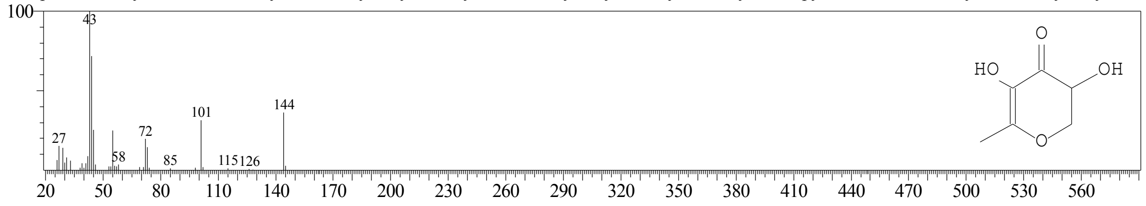

Hit#:4 Entry:42586 Library:Wiley9.lib
SI:94 Formula:C6H8O4 CAS:28564-83-2 MolWeight:144 RetIndex:0
CompName:4H-Pyran-4-one, 2,3-dihydro-3,5-dihydroxy-6-methyl- (CAS) $$ 3,5-DIHYDROXY-2-METHYL-5,6-DIHYDROPYRAN-4-ONE $$ 4H-PYRAN-4

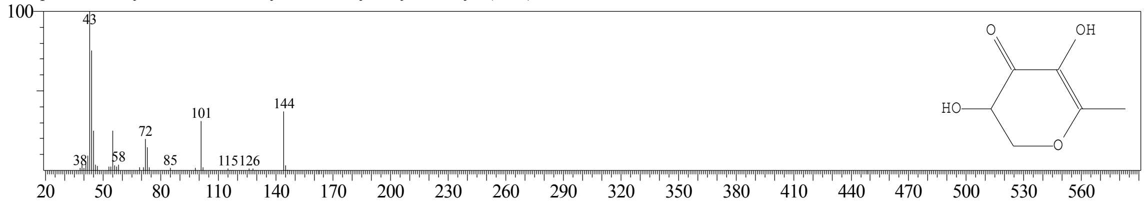

Hit#:5 Entry:42592 Library:Wiley9.lib
SI:90 Formula:C6H8O4 CAS:28564-83-2 MolWeight:144 RetIndex:0
CompName:2,3-Dihydro-3,5-dihydroxy-6-methyl-4H-pyran-4-one

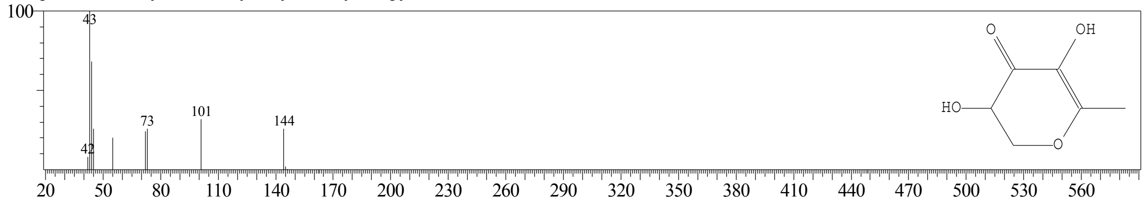

<< Target >>
Line#:9 R.Time:8.700(Scan#:741) MassPeaks:306
RawMode:Averaged 8.695-8.705(740-742) BasePeak:44.00(66150)
BG Mode:Calc. from Peak Group 1 - Event 1

Target Spectrum

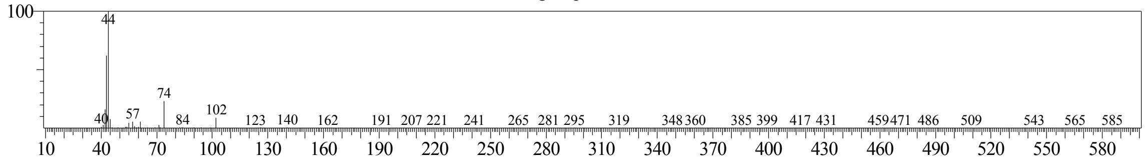

Hit#:1 Entry:8996 Library:Wiley9.lib
SI:94 Formula:C4H6O3 CAS:5469-16-9 MolWeight:102 RetIndex:0
CompName:2(3H)-Furanone, dihydro-4-hydroxy- $$ N-[2-(3,5-DIMETHOXY-PHENYL)-2-METHYL-PROPYL]-N-ETHYL-ACETAMIDE $$ 4-Hydroxydihyc

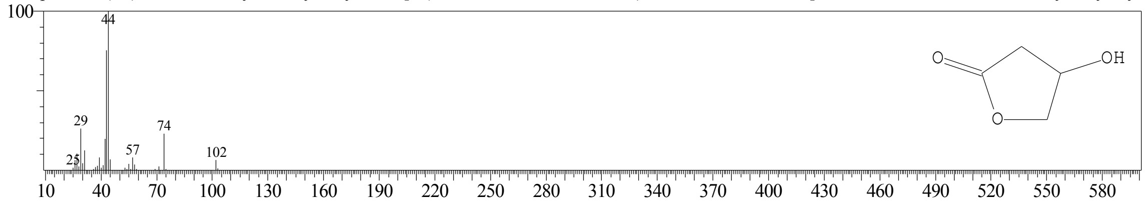

Hit#:2 Entry:2211 Library:NIST11.lib
SI:94 Formula:C4H6O3 CAS:5469-16-9 MolWeight:102 RetIndex:1013
CompName:2(3H)-Furanone, dihydro-4-hydroxy- $$ 4-Hydroxydihydro-2(3H)-furanone # $$ Dihydro-4-hydroxy-2-(3H)-furanone $$

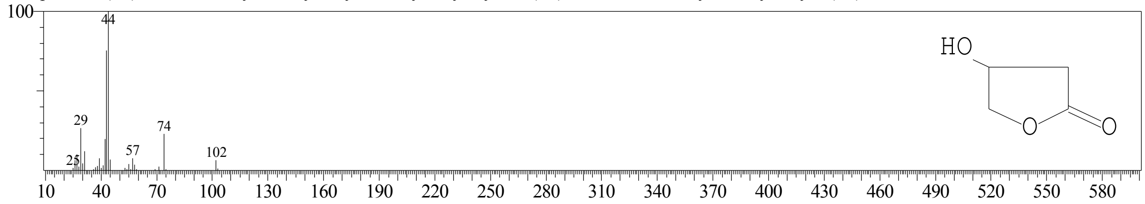

Hit#:3 Entry:9000 Library:Wiley9.lib
SI:92 Formula:C4H6O3 CAS:0-00-0 MolWeight:102 RetIndex:0
CompName:2 - hydroxy - butanedial $$ 2 - hydroxy - butanedialdehyde

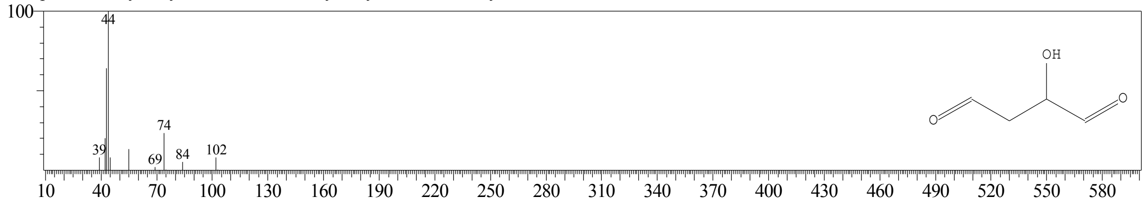

Hit#:4 Entry:9001 Library:Wiley9.lib
SI:88 Formula:C4H6O3 CAS:0-00-0 MolWeight:102 RetIndex:0
CompName:2 - hydroxy - butanedial $$ 2 - hydroxy - butanedialdehyde

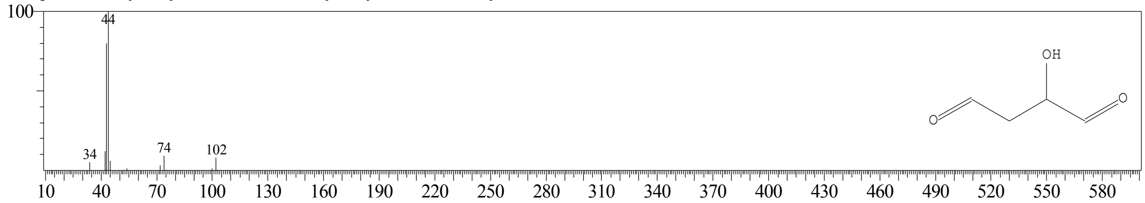

Hit#:5 Entry:1955 Library:Wiley9.lib
SI:88 Formula:C3H6O2 CAS:556-52-5 MolWeight:74 RetIndex:0
CompName:Oxiranemethanol (CAS) $$ Glycidol (CAS) $$ Glycide $$ Glycidyl alcohol $$ Epihydrin alcohol $$ Allyl alcohol oxide $$ 2,3-Epoxy-1-propanol $

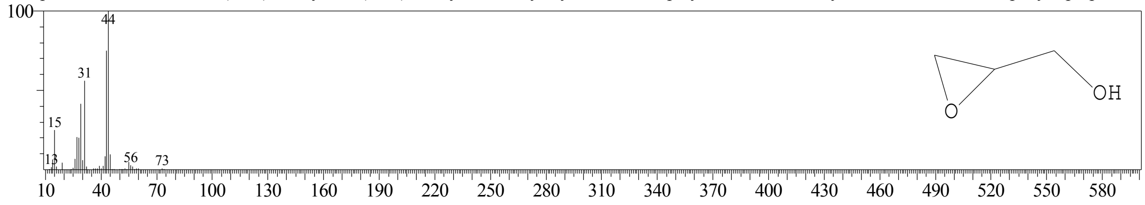

<< Target >>
Line#:13   R.Time:9.815(Scan#:964) MassPeaks:299
RawMode:Averaged 9.810-9.820(963-965) BasePeak:97.05(375927)
BG Mode:Calc. from Peak Group 1 - Event 1

Target Spectrum

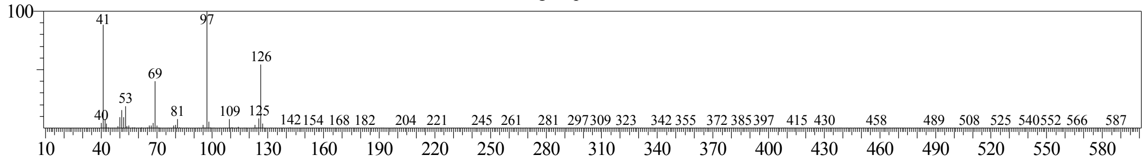

Hit#:1 Entry:6369 Library:NIST11.lib
SI:94 Formula:C6H6O3 CAS:67-47-0 MolWeight:126 RetIndex:1163
CompName:5-Hydroxymethylfurfural $$ 2-Furancarboxaldehyde, 5-(hydroxymethyl)- $$ 2-Furaldehyde, 5-(hydroxymethyl)- $$ HMF $$ 5-(Hyddroxymethyl)fu

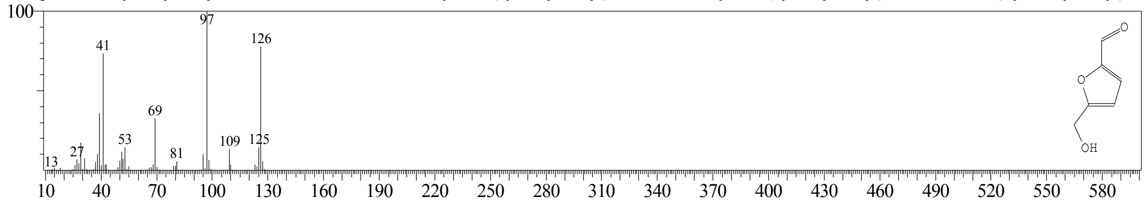

Hit#:2 Entry:22986 Library:Wiley9.lib
SI:93 Formula:C6H6O3 CAS:67-47-0 MolWeight:126 RetIndex:0
CompName:2-FURANCARBOXALDEHYDE, 5-(HYDROXYMETHYL)- $$ 2-FURALDEHYDE, 5- (HYDROXYMETHYL)- $$ 2-FURALDEHYDE, 5-(HY

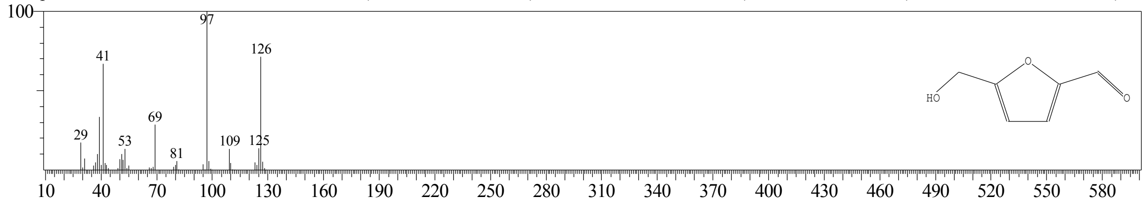

Hit#:3 Entry:22987 Library:Wiley9.lib
SI:89 Formula:C6H6O3 CAS:67-47-0 MolWeight:126 RetIndex:0
CompName:2-Furancarboxaldehyde, 5-(hydroxymethyl)- (CAS) $$ HMF $$ 5-Oxymethylfurfurole $$ Hydroxymethylfurfurole $$ 5-(Hydroxymethyl)furfural $$

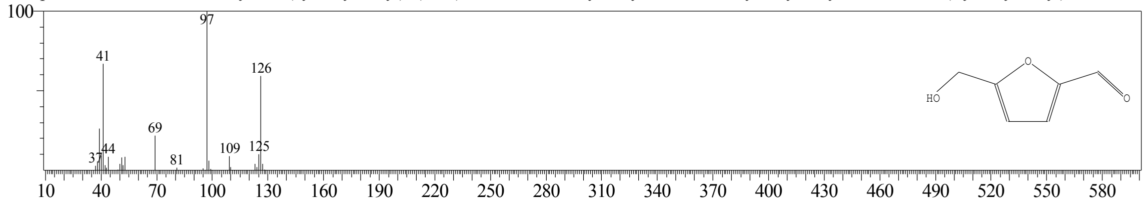

Hit#:4 Entry:22988 Library:Wiley9.lib
SI:86 Formula:C6H6O3 CAS:67-47-0 MolWeight:126 RetIndex:0
CompName:2-Furancarboxaldehyde, 5-(hydroxymethyl)- (CAS) $$ HMF $$ 5-Oxymethylfurfurole $$ Hydroxymethylfurfurole $$ 5-(Hydroxymethyl)furfural $$

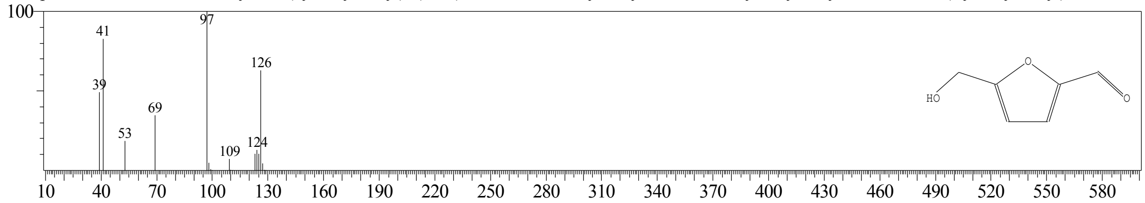

Hit#:5 Entry:6505 Library:NIST11.lib
SI:85 Formula:C8H14O CAS:17325-90-5 MolWeight:126 RetIndex:915
CompName:4-Hexen-3-one, 4,5-dimethyl- $$ 4,5-Dimethyl-4-hexen-3-one # $$

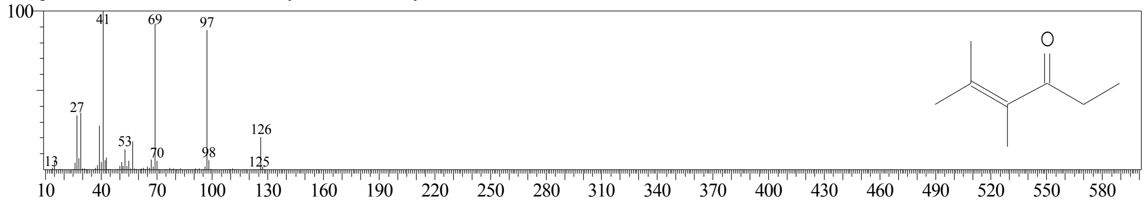

<< Target >>
Line#:14 R.Time:10.115(Scan#:1024) MassPeaks:322
RawMode:Averaged 10.110-10.120(1023-1025) BasePeak:43.00(502592)
BG Mode:Calc. from Peak Group 1 - Event 1

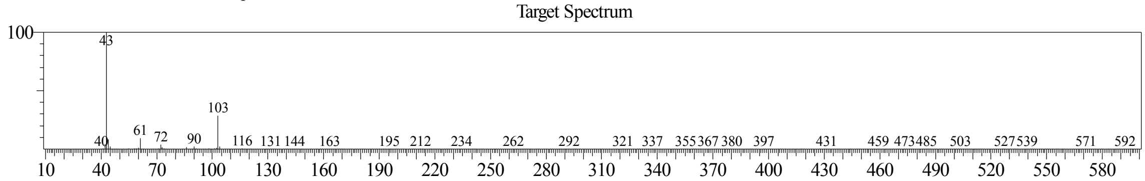

Hit#:1 Entry:8833 Library:NIST11.lib
SI:92 Formula:C5H10O4 CAS:106-61-6 MolWeight:134 RetIndex:1091
CompName:1,2,3-Propanetriol, 1-acetate $$ Acetin, 1-mono- $$ .alpha.-Monoacetin $$ Glycerol .alpha.-monoacetate $$ 1-Monoacetin $$ 2,3-Dihydroxypropyl

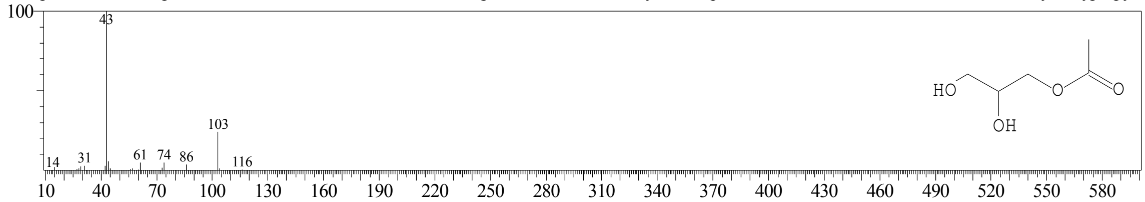

Hit#:2 Entry:30510 Library:Wiley9.lib
SI:92 Formula:C5H10O4 CAS:26446-35-5 MolWeight:134 RetIndex:0
CompName:1,2,3-Propanetriol, monoacetate $$ Acetin, mono- $$ Acetin $$ Acetoglyceride $$ Acetyl monoglyceride $$ Glycerin monoacetate $$ Glycerol acetat

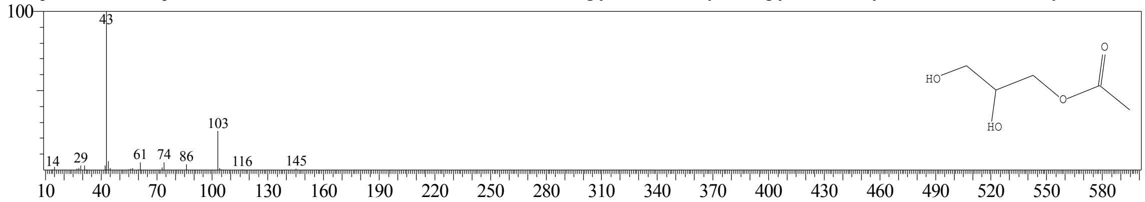

Hit#:3 Entry:30508 Library:Wiley9.lib
SI:92 Formula:C5H10O4 CAS:106-61-6 MolWeight:134 RetIndex:0
CompName:1,2,3-Propanetriol, 1-acetate (CAS) $$ 1-ACETOXY-2,3-DIHYDROXYPROPANE $$ 1-Monoacetin $$ Acetin, 1-mono- $$ .alpha.-Monoacetin $$

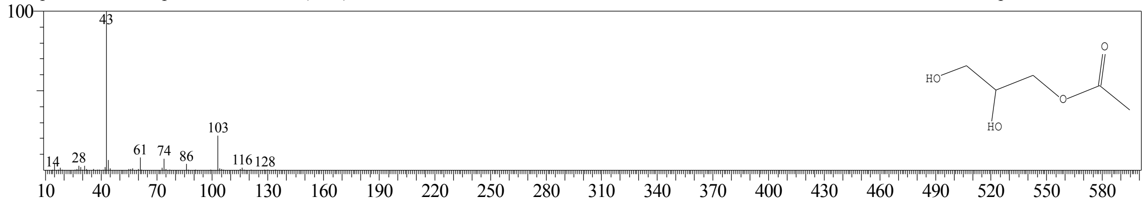

Hit#:4 Entry:93203 Library:Wiley9.lib
SI:91 Formula:C7H12O5 CAS:25395-31-7 MolWeight:176 RetIndex:0
CompName:1,2,3-Propanetriol, diacetate (CAS) $$ Diacetin $$ Acetin, di- $$ Diacetylglycerol $$ Glycerin diacetate $$ Glyceryl diacetate $$ Glycerol diacetate

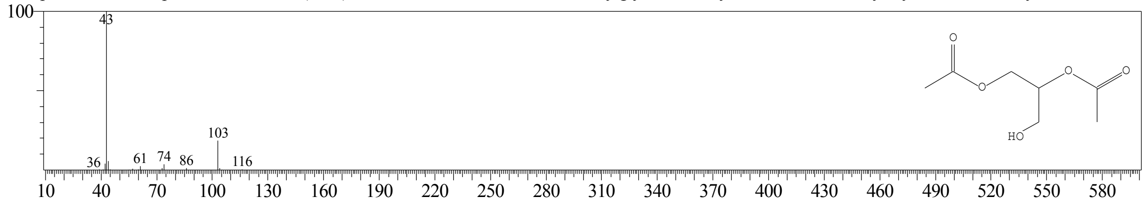

Hit#:5 Entry:30509 Library:Wiley9.lib
SI:91 Formula:C5H10O4 CAS:26446-35-5 MolWeight:134 RetIndex:0
CompName:1,2,3-Propanetriol, monoacetate $$ Acetin, mono- $$ Acetin $$ Acetoglyceride $$ Acetyl monoglyceride $$ Glycerin monoacetate $$ Glycerol acetat

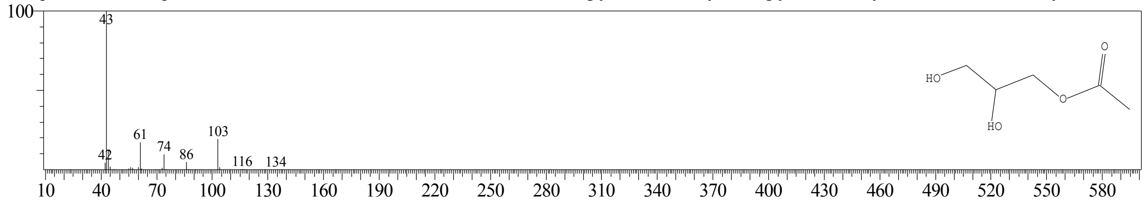

<< Target >>
Line#:25 R.Time:19.765(Scan#:2954) MassPeaks:314
RawMode:Averaged 19.760-19.770(2953-2955) BasePeak:73.05(20385)
BG Mode:Calc. from Peak Group 1 - Event 1

Target Spectrum

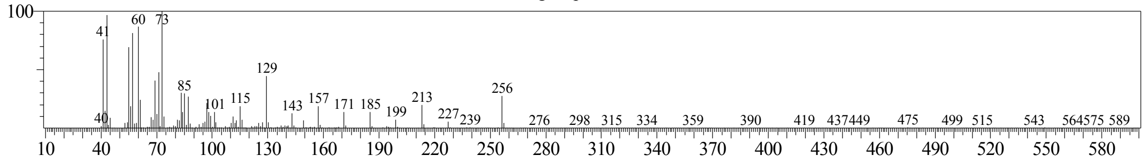

Hit#:1 Entry:274466 Library:Wiley9.lib
SI:94 Formula:C16H32O2 CAS:57-10-3 MolWeight:256 RetIndex:0
CompName:Hexadecanoic acid (CAS) $$ Palmitic acid $$ Palmitinic acid $$ n-Hexadecoic acid $$ n-Hexadecanoic acid $$ Pentadecanecarboxylic acid $$ 1-Pe

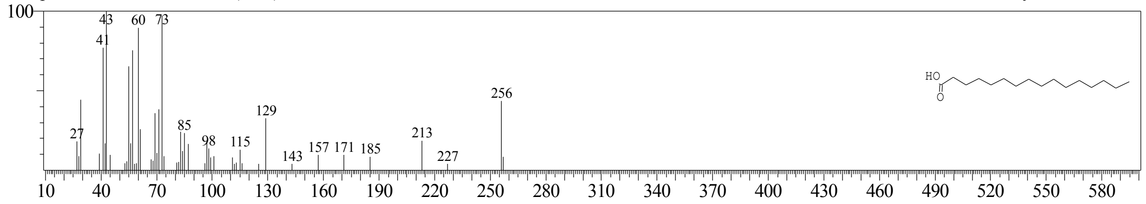

Hit#:2 Entry:241462 Library:Wiley9.lib
SI:93 Formula:C15H30O2 CAS:1002-84-2 MolWeight:242 RetIndex:0
CompName:Pentadecanoic acid (CAS) $$ Pentadecylic acid $$ n-Pentadecanoic acid $$ n-Pentadecylic acid $$ Pentadecyclic acid $$ PENTADECANSAEURE

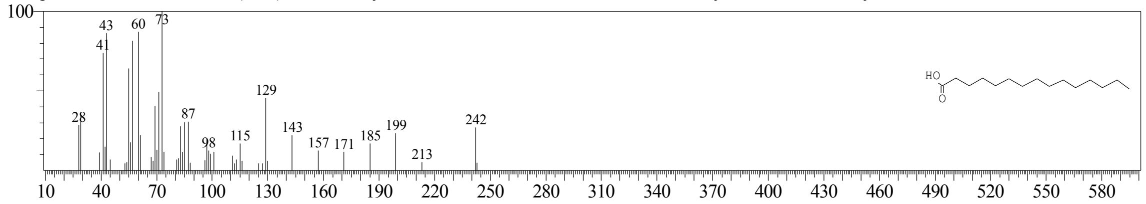

Hit#:3 Entry:73851 Library:NIST11.lib
SI:93 Formula:C15H30O2 CAS:1002-84-2 MolWeight:242 RetIndex:1869
CompName:Pentadecanoic acid $$ Pentadecylic acid $$ n-Pentadecanoic acid $$ n-Pentadecylic acid $$

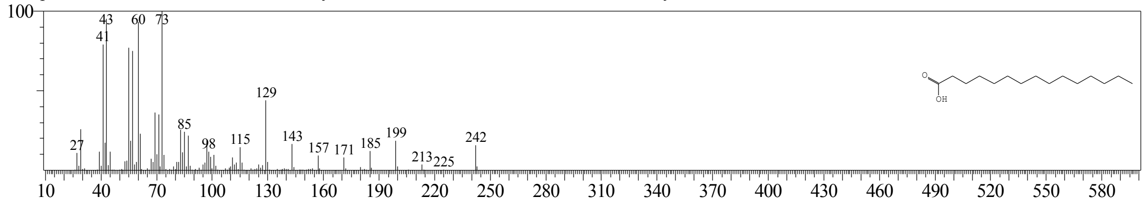

Hit#:4 Entry:274464 Library:Wiley9.lib
SI:92 Formula:C16H32O2 CAS:57-10-3 MolWeight:256 RetIndex:0
CompName:Hexadecanoic acid (CAS) $$ Palmitic acid $$ Palmitinic acid $$ n-Hexadecoic acid $$ n-Hexadecanoic acid $$ Pentadecanecarboxylic acid $$ 1-Pe

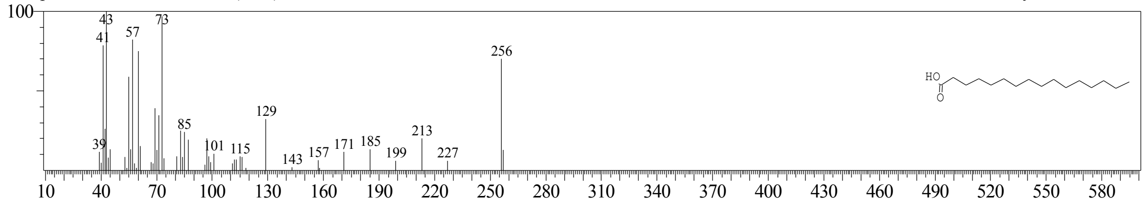

Hit#:5 Entry:106158 Library:NIST11.lib
SI:91 Formula:C18H36O2 CAS:57-11-4 MolWeight:284 RetIndex:2167
CompName:Octadecanoic acid $$ Stearic acid $$ n-Octadecanoic acid $$ Humko Industrene R $$ Hydrofol Acid 150 $$ Hystrene S-97 $$ Hystrene T-70 $$ Hys

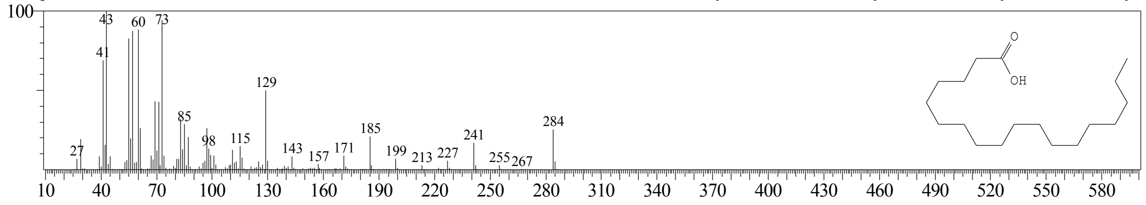

<< Target >>
Line#:26 R.Time:22.325(Scan#:3466) MassPeaks:339
RawMode:Averaged 22.320-22.330(3465-3467) BasePeak:67.05(16926)
BG Mode:Calc. from Peak Group 1 - Event 1

Target Spectrum

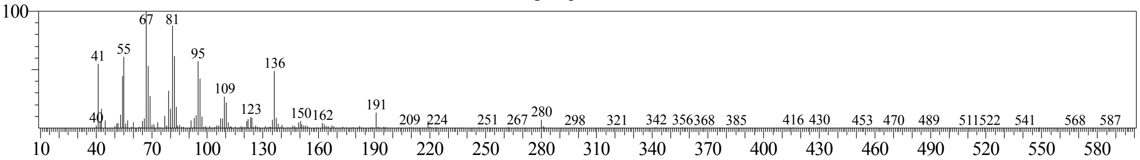

Hit#:1 Entry:102816 Library:NIST11.lib
SI:91 Formula:C18H32O2 CAS:60-33-3 MolWeight:280 RetIndex:2183
CompName:9,12-Octadecadienoic acid (Z,Z)- $$ cis-9,cis-12-Octadecadienoic acid $$ cis,cis-Linoleic acid $$ Grape seed oil $$ Linoleic $$ Linoleic acid $$ Li

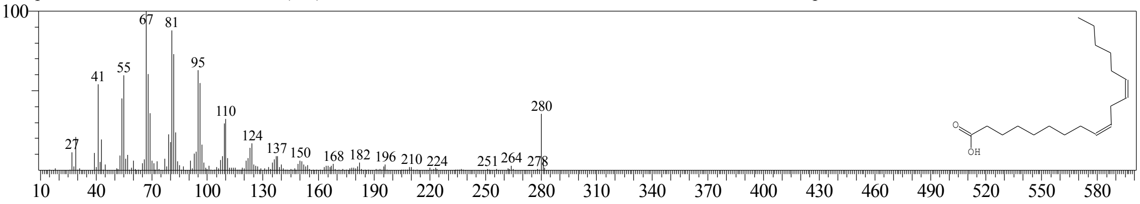

Hit#:2 Entry:361853 Library:Wiley9.lib
SI:90 Formula:C19H34O2 CAS:2462-85-3 MolWeight:294 RetIndex:0
CompName:9,12-Octadecadienoic acid, methyl ester $$ OCTADECA-9,12-DIENOIC ACID METHYL ESTER $$ Methyl (9E,12E)-9,12-octadecadienoate $$ M

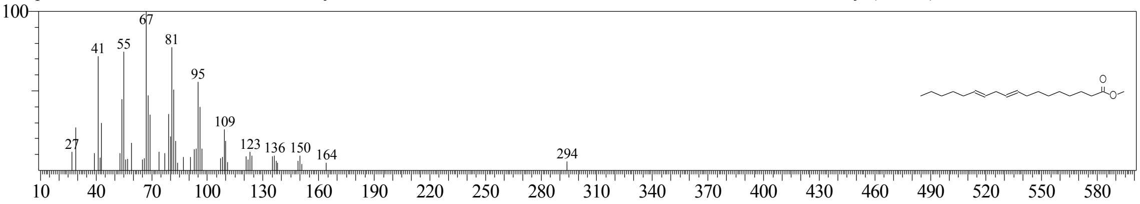

Hit#:3 Entry:113947 Library:NIST11.lib
SI:90 Formula:C19H34O2 CAS:2462-85-3 MolWeight:294 RetIndex:2093
CompName:9,12-Octadecadienoic acid, methyl ester $$ 9,12-Octadecenoic acid, methyl ester $$ Methyl octadeca-9,12-dienoate $$ Methyl 9,12-octadecadienoa

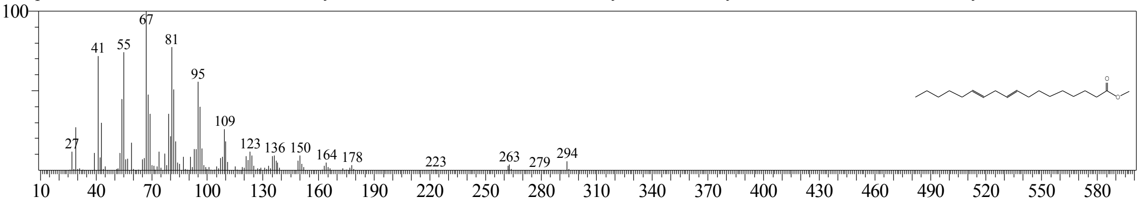

Hit#:4 Entry:81243 Library:NIST11.lib
SI:89 Formula:C16H28O2 CAS:123-69-3 MolWeight:252 RetIndex:2246
CompName:Oxacycloheptadec-8-en-2-one, (8Z) $$ Ambrettolid $$ Ambrettolide $$ Musk ambrette $$ Musk ambrette, natural $$ Musk natural $$ 7-Hexadecen

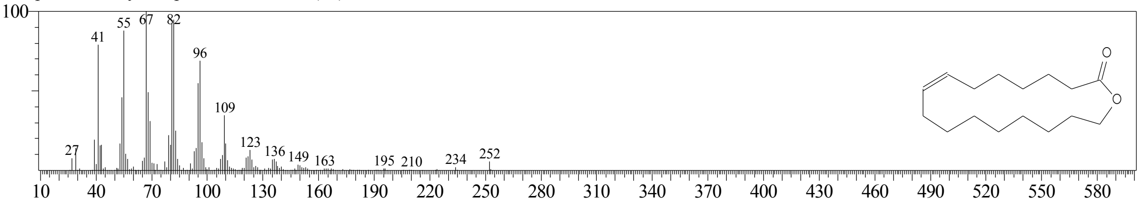

Hit#:5 Entry:265048 Library:Wiley9.lib
SI:89 Formula:C16H28O2 CAS:123-69-3 MolWeight:252 RetIndex:0
CompName:Oxacycloheptadec-8-en-2-one (CAS) $$ Ambrettolide $$ Ambrettolid $$ Musk natural $$ Musk ambrette $$ Musk ambrette, natural $$ 7-Hexadece

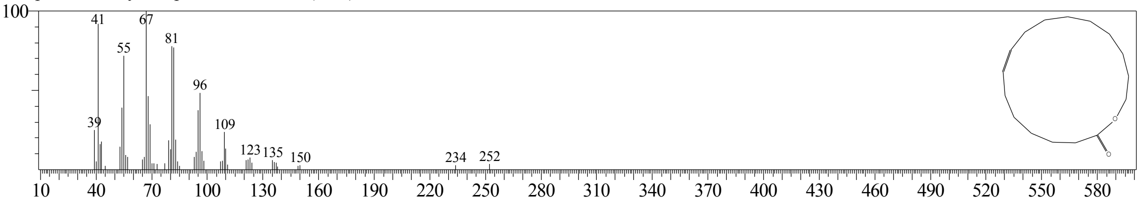

<< Target >>
Line#:27 R.Time:22.415(Scan#:3484) MassPeaks:329
RawMode:Averaged 22.410-22.420(3483-3485) BasePeak:79.10(10860)
BG Mode:Calc. from Peak Group 1 - Event 1

Target Spectrum

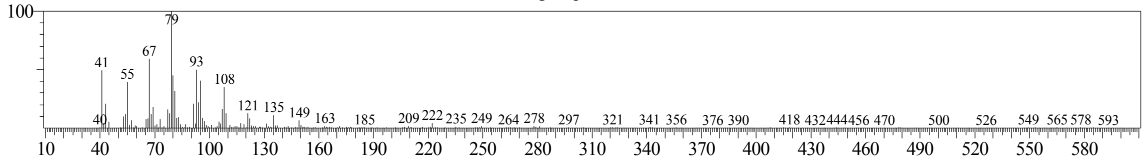

Hit#:1 Entry:101390 Library:NIST11.lib
SI:95 Formula:C18H30O2 CAS:463-40-1 MolWeight:278 RetIndex:2191
CompName:9,12,15-Octadecatrienoic acid, (Z,Z,Z)- $$ Linolenic acid $$ .alpha.-Linolenic acid $$ All-cis-9,12,15-Octadecatrienoic acid $$ cis,cis,cis-9,12,15-

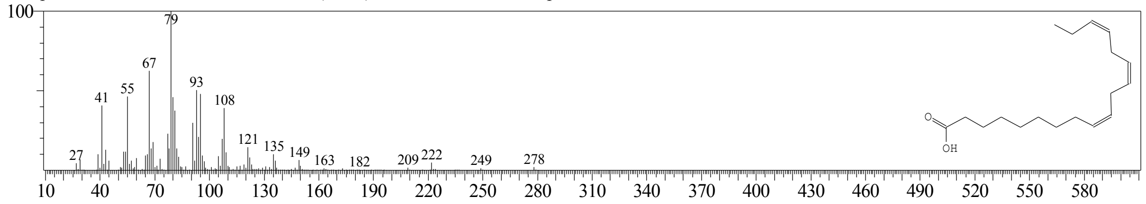

Hit#:2 Entry:357250 Library:Wiley9.lib
SI:93 Formula:C19H32O2 CAS:301-00-8 MolWeight:292 RetIndex:0
CompName:9,12,15-Octadecatrienoic acid, methyl ester, (Z,Z,Z)- (CAS) $$ Methyl linolenate $$ Linolenic acid methyl ester $$ Linolenic acid, methyl ester $$

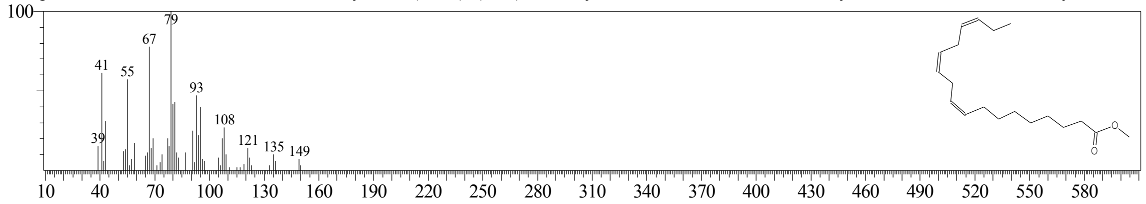

Hit#:3 Entry:417437 Library:Wiley9.lib
SI:92 Formula:C21H36O2 CAS:55682-88-7 MolWeight:320 RetIndex:0
CompName:11,14,17-Eicosatrienoic acid, methyl ester (CAS) $$ METHYL-11,14,17-EICOSATRIENOATE $$ Methyl (11E,14E,17E)-11,14,17-icosatrienoate

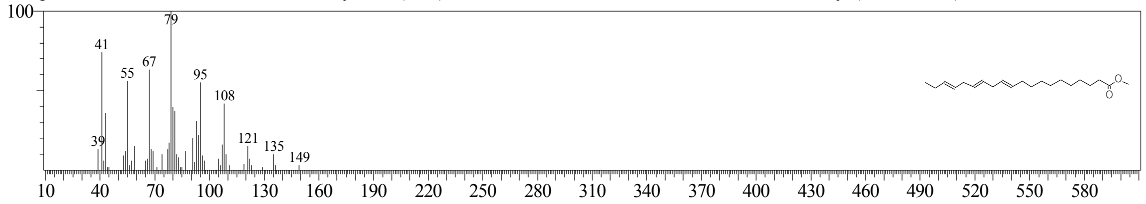

Hit#:4 Entry:357254 Library:Wiley9.lib
SI:91 Formula:C19H32O2 CAS:301-00-8 MolWeight:292 RetIndex:0
CompName:9,12,15-Octadecatrienoic acid, methyl ester, (Z,Z,Z)- (CAS) $$ Methyl linolenate $$ Linolenic acid methyl ester $$ Linolenic acid, methyl ester $$

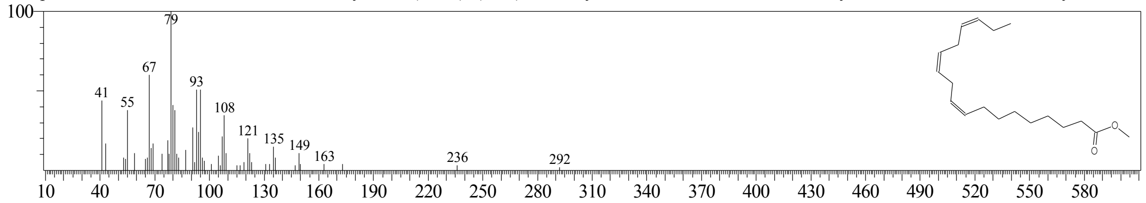

Hit#:5 Entry:90503 Library:NIST11.lib
SI:91 Formula:C18H32O CAS:506-44-5 MolWeight:264 RetIndex:2077
CompName:9,12,15-Octadecatrien-1-ol, (Z,Z,Z)- $$ (9E,12E,15E)-9,12,15-Octadecatrien-1-ol # $$ (Z,Z,Z)-9,12,15-Octadecatrien-1-ol $$

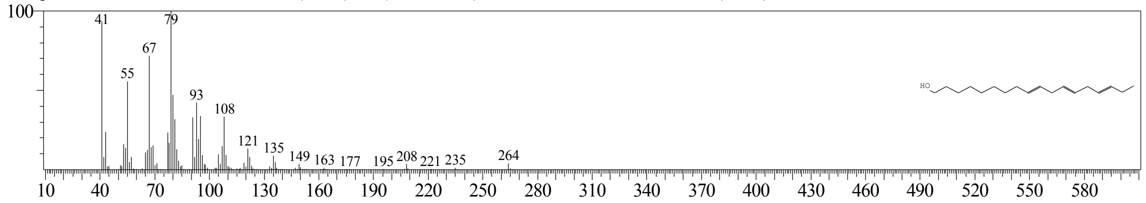

<< Target >>
Line#:28 R.Time:23.055(Scan#:3612) MassPeaks:344
RawMode:Averaged 23.050-23.060(3611-3613) BasePeak:59.05(180775)
BG Mode:Calc. from Peak Group 1 - Event 1

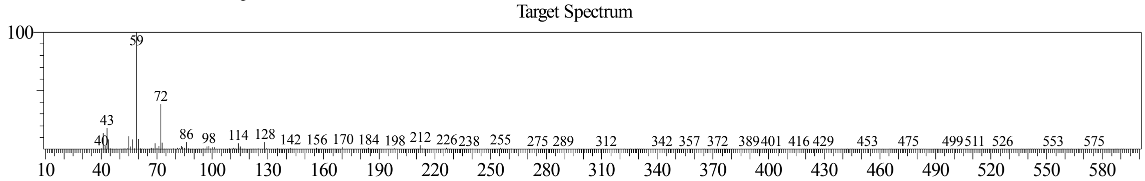

Hit#:1 Entry:271776 Library:Wiley9.lib
SI:97 Formula:C16H33NO CAS:629-54-9 MolWeight:255 RetIndex:0
CompName:Hexadecanamide (CAS) $$ Amide 16 $$ Amide HPL $$ Cetyl amide $$ Palmitamide $$ Palmityl amide $$ Palmitic amide $$ n-Hexadecanamide $$

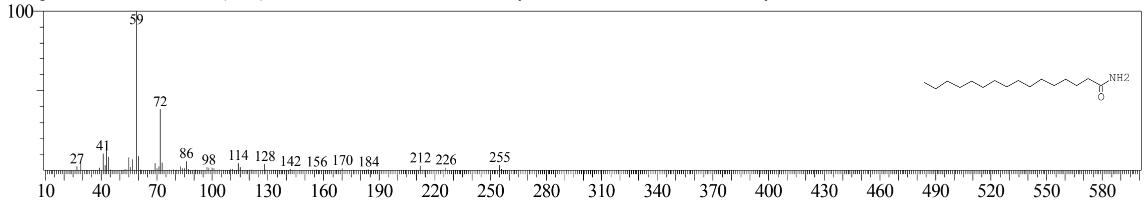

Hit#:2 Entry:83422 Library:NIST11.lib
SI:96 Formula:C16H33NO CAS:629-54-9 MolWeight:255 RetIndex:2021
CompName:Hexadecanamide $$ Palmitamide $$ n-Hexadecanamide $$ Amide HPL $$ Amide 16 $$ Cetyl amide $$ Palmitic acid amide $$ Palmitic amide $$ Pa

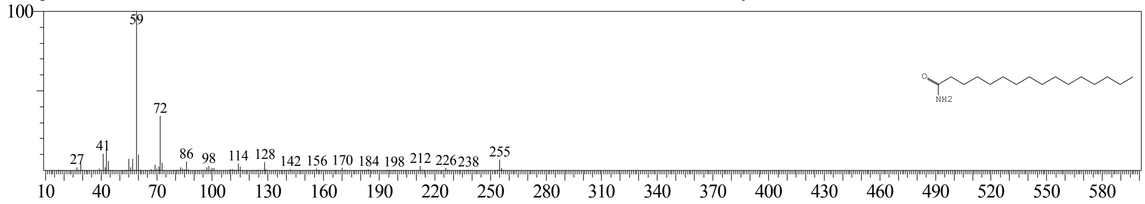

Hit#:3 Entry:43281 Library:NIST11.lib
SI:94 Formula:C12H25NO CAS:1120-16-7 MolWeight:199 RetIndex:1623
CompName:Dodecanamide $$ Lauramide $$ Amide KK $$ Dodecamide $$ Dodecylamide $$ Lauric amide $$ Lauryl amide $$ Lauroylamide $$ Diamide Y $$ n-

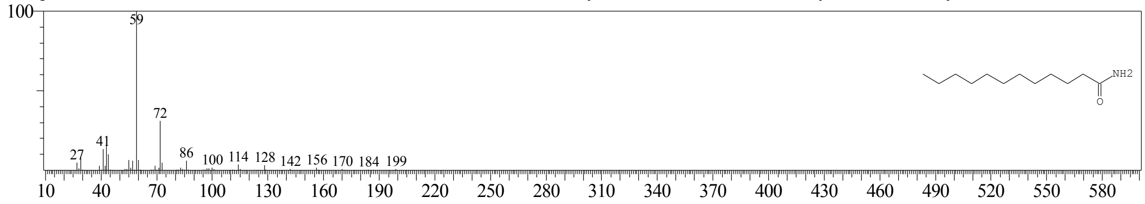

Hit#:4 Entry:62912 Library:NIST11.lib
SI:94 Formula:C14H29NO CAS:638-58-4 MolWeight:227 RetIndex:1822
CompName:Tetradecanamide $$ Myristamide $$ Myristic acid amide $$ Myristic amide $$ Tetradecylamide $$

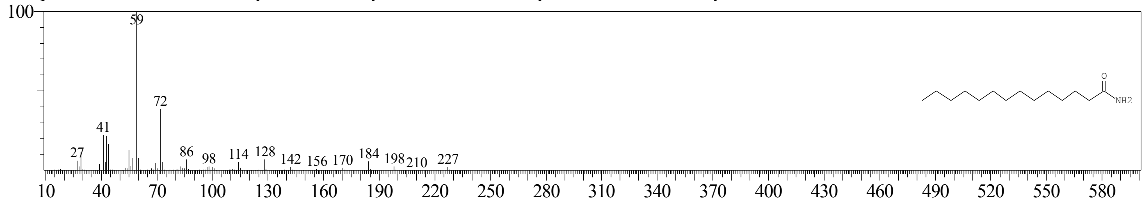

Hit#:5 Entry:105140 Library:NIST11.lib
SI:94 Formula:C18H37NO CAS:124-26-5 MolWeight:283 RetIndex:2220
CompName:Octadecanamide $$ Stearamide $$ Adogen 42 $$ Octadecamide $$ Octadecylamide $$ Stearic acid amide $$ Stearic amide $$ Stearoylamide $$ Stea

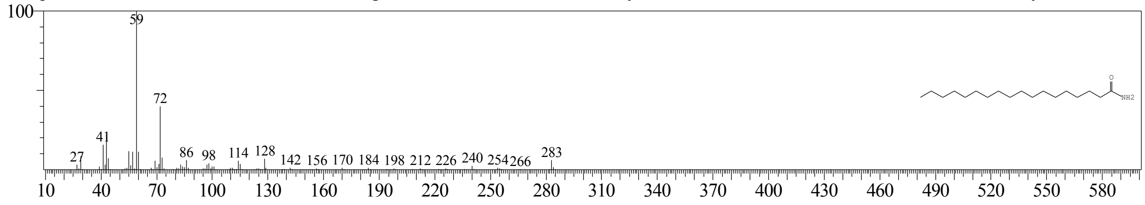

<< Target >>
Line#:29 R.Time:25.890(Scan#:4179) MassPeaks:358
RawMode:Averaged 25.885-25.895(4178-4180) BasePeak:59.05(98623)
BG Mode:Calc. from Peak Group 1 - Event 1

Target Spectrum

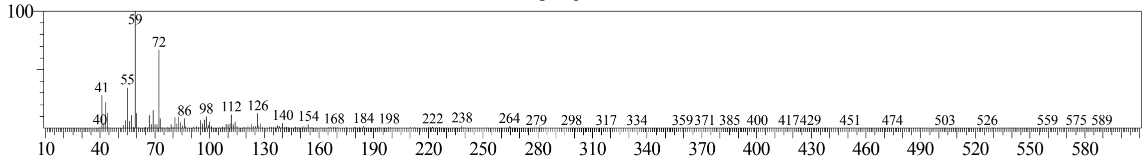

Hit#:1 Entry:332229 Library:Wiley9.lib
SI:95 Formula:C18H35NO CAS:3322-62-1 MolWeight:281 RetIndex:0
CompName:9-Octadecenamide (CAS) $$ 9-OCTADECENYLAMIDE $$ Armid ow $$ Armoslip cp $$ (9E)-9-Octadecenamide $$ 9 - octadecenamide $$ (9E)-9-

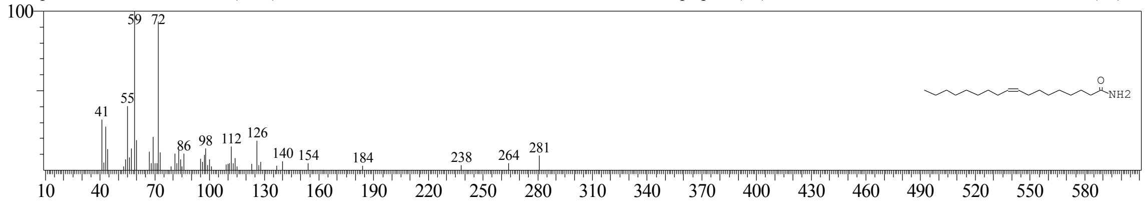

Hit#:2 Entry:332232 Library:Wiley9.lib
SI:95 Formula:C18H35NO CAS:301-02-0 MolWeight:281 RetIndex:0
CompName:9-OCTADECENAMIDE $$ 9-OCTADECENAMIDE, (Z)- $$ (9Z)-9-OCTADECENAMIDE # $$ (9Z)-9-OCTADECENAMIDE $$ (9Z)-9-OCTAD

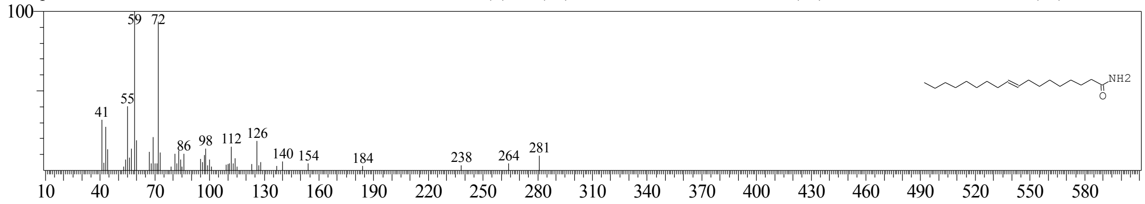

Hit#:3 Entry:103511 Library:NIST11.lib
SI:91 Formula:C18H35NO CAS:301-02-0 MolWeight:281 RetIndex:2228
CompName:9-Octadecenamide, (Z)- $$ Adogen 73 $$ Oleamide $$ Oleic acid amide $$ Oleyl amide $$ Slip-eze $$ Armoslip CP $$ Crodamide O $$ Crodamide

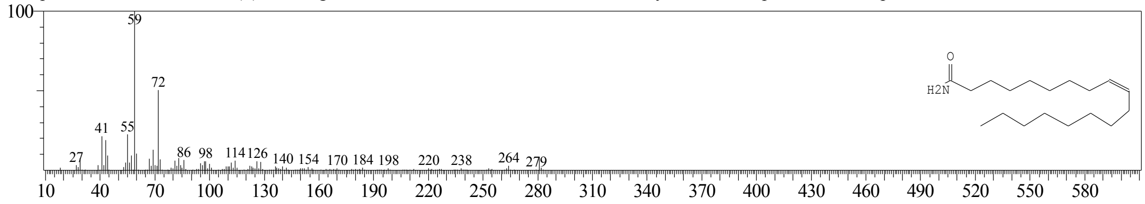

Hit#:4 Entry:332234 Library:Wiley9.lib
SI:88 Formula:C18H35NO CAS:301-02-0 MolWeight:281 RetIndex:0
CompName:9-Octadecenamide, (Z)- (CAS) $$ OLEOAMIDE $$ OELIC ACID AMIDE $$ Oleamide $$ Adogen 73 $$ Oleylamide $$ Slip-ezeCI) $$ Oleic acid a

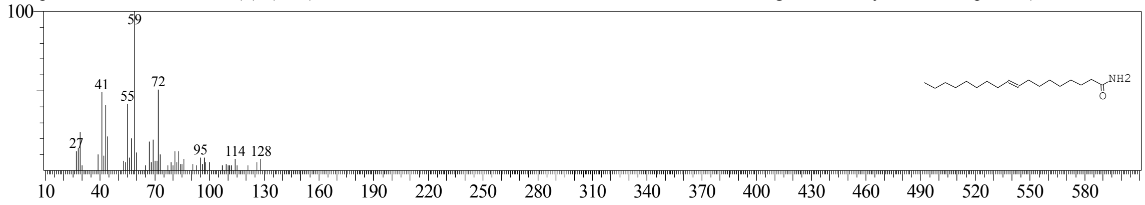

Hit#:5 Entry:146739 Library:NIST11.lib
SI:84 Formula:C22H43NO CAS:112-84-5 MolWeight:337 RetIndex:2625
CompName:13-Docosenamide, (Z)- $$ Erucylamide $$ Erucyl amide $$ (Z)-13-Docosenamide $$ 13-Docosenamide, cis- $$ Armid E $$ cis-13-Docosenamide $

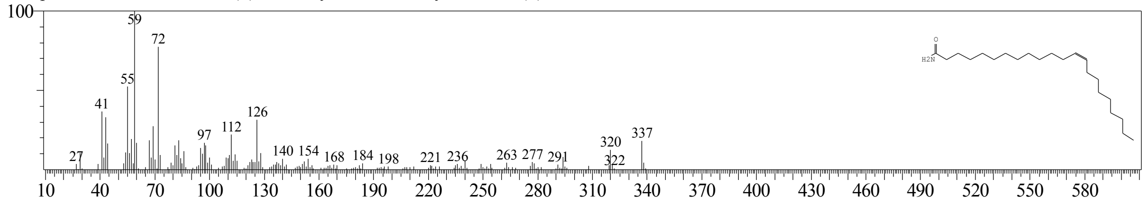

<< Target >>
Line#:30 R.Time:25.990(Scan#:4199) MassPeaks:371
RawMode:Averaged 25.985-25.995(4198-4200) BasePeak:59.05(55575)
BG Mode:Calc. from Peak Group 1 - Event 1

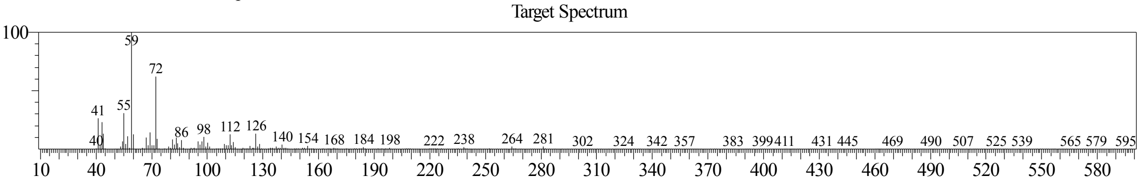

Hit#:1 Entry:332229 Library:Wiley9.lib
SI:94 Formula:C18H35NO CAS:3322-62-1 MolWeight:281 RetIndex:0
CompName:9-Octadecenamide (CAS) $$ 9-OCTADECENYLAMIDE $$ Armid ow $$ Armoslip cp $$ (9E)-9-Octadecenamide $$ 9 - octadecenamide $$ (9E)-9-

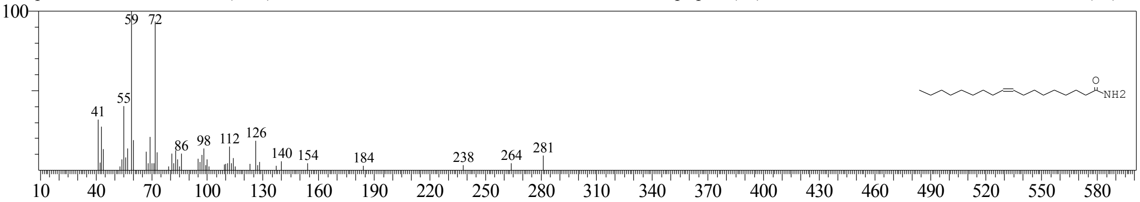

Hit#:2 Entry:332232 Library:Wiley9.lib
SI:94 Formula:C18H35NO CAS:301-02-0 MolWeight:281 RetIndex:0
CompName:9-OCTADECENAMIDE $$ 9-OCTADECENAMIDE, (Z)- $$ (9Z)-9-OCTADECENAMIDE # $$ (9Z)-9-OCTADECENAMIDE $$ (9Z)-9-OCTAD

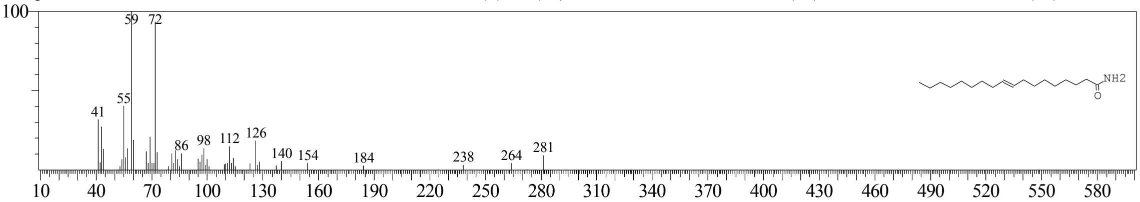

Hit#:3 Entry:103511 Library:NIST11.lib
SI:92 Formula:C18H35NO CAS:301-02-0 MolWeight:281 RetIndex:2228
CompName:9-Octadecenamide, (Z)- $$ Adogen 73 $$ Oleamide $$ Oleic acid amide $$ Oleyl amide $$ Slip-eze $$ Armoslip CP $$ Crodamide O $$ Crodamide

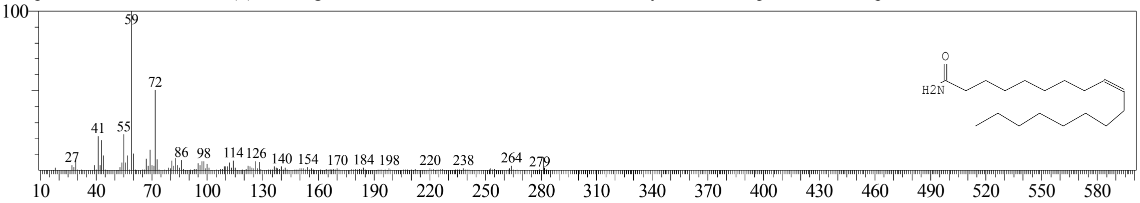

Hit#:4 Entry:332234 Library:Wiley9.lib
SI:88 Formula:C18H35NO CAS:301-02-0 MolWeight:281 RetIndex:0
CompName:9-Octadecenamide, (Z)- (CAS) $$ OLEOAMIDE $$ OELIC ACID AMIDE $$ Oleamide $$ Adogen 73 $$ Oleylamide $$ Slip-ezeCI) $$ Oleic acid a

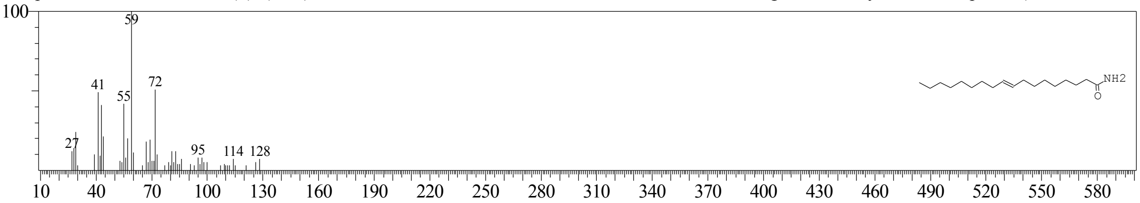

Hit#:5 Entry:146739 Library:NIST11.lib
SI:83 Formula:C22H43NO CAS:112-84-5 MolWeight:337 RetIndex:2625
CompName:13-Docosenamide, (Z)- $$ Erucylamide $$ Erucyl amide $$ (Z)-13-Docosenamide $$ 13-Docosenamide, cis- $$ Armid E $$ cis-13-Docosenamide $

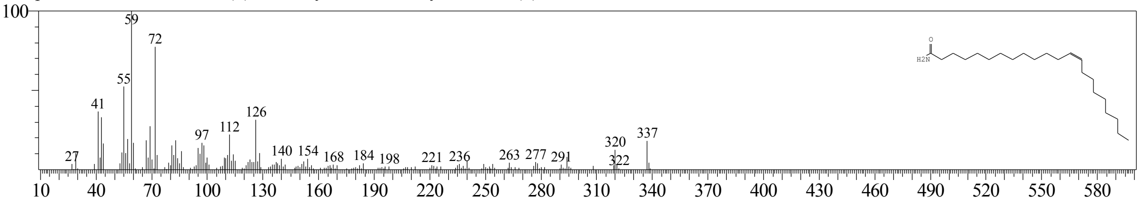

<< Target >>
Line#:31 R.Time:26.065(Scan#:4214) MassPeaks:363
RawMode:Averaged 26.060-26.070(4213-4215) BasePeak:59.05(64075)
BG Mode:Calc. from Peak Group 1 - Event 1

Target Spectrum

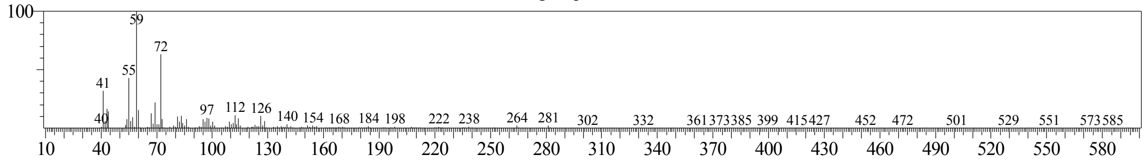

Hit#:1 Entry:332229 Library:Wiley9.lib
SI:95 Formula:C18H35NO CAS:3322-62-1 MolWeight:281 RetIndex:0
CompName:9-Octadecenamide (CAS) $$ 9-OCTADECENYLAMIDE $$ Armid ow $$ Armoslip cp $$ (9E)-9-Octadecenamide $$ 9 - octadecenamide $$ (9E)-9-

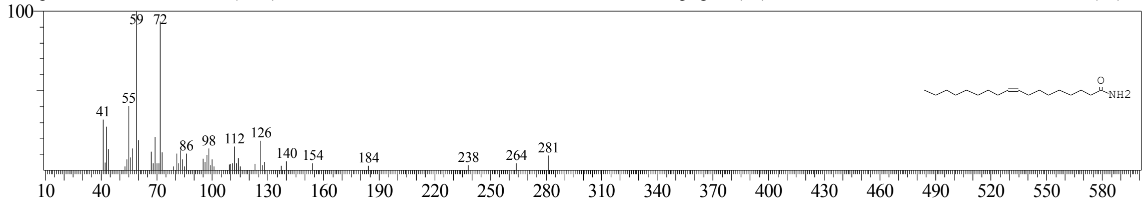

Hit#:2 Entry:332232 Library:Wiley9.lib
SI:95 Formula:C18H35NO CAS:301-02-0 MolWeight:281 RetIndex:0
CompName:9-OCTADECENAMIDE $$ 9-OCTADECENAMIDE, (Z)- $$ (9Z)-9-OCTADECENAMIDE # $$ (9Z)-9-OCTADECENAMIDE $$ (9Z)-9-OCTAD

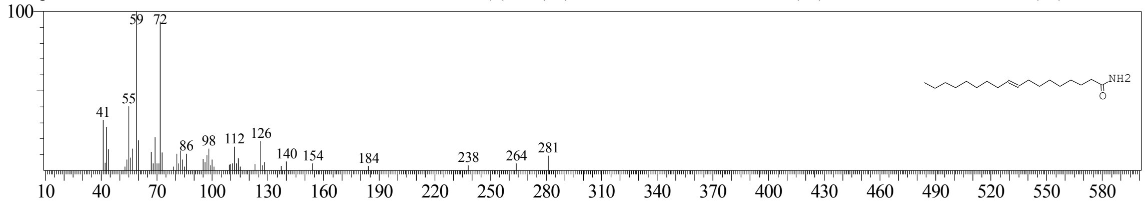

Hit#:3 Entry:103511 Library:NIST11.lib
SI:90 Formula:C18H35NO CAS:301-02-0 MolWeight:281 RetIndex:2228
CompName:9-Octadecenamide, (Z)- $$ Adogen 73 $$ Oleamide $$ Oleic acid amide $$ Oleyl amide $$ Slip-eze $$ Armoslip CP $$ Crodamide O $$ Crodamide

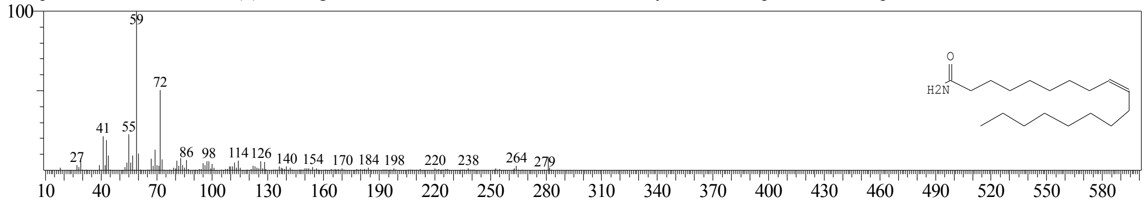

Hit#:4 Entry:332234 Library:Wiley9.lib
SI:89 Formula:C18H35NO CAS:301-02-0 MolWeight:281 RetIndex:0
CompName:9-Octadecenamide, (Z)- (CAS) $$ OLEOAMIDE $$ OELIC ACID AMIDE $$ Oleamide $$ Adogen 73 $$ Oleylamide $$ Slip-ezeCI) $$ Oleic acid a

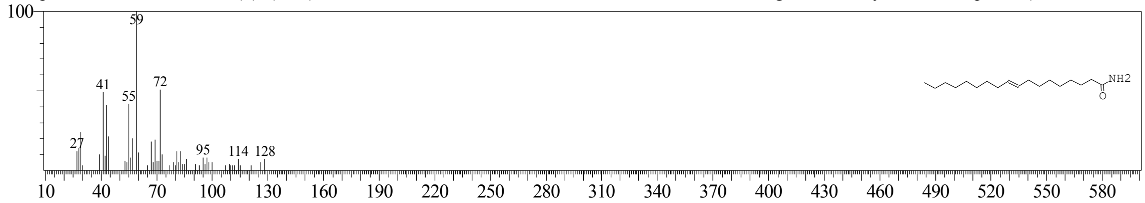

Hit#:5 Entry:146739 Library:NIST11.lib
SI:85 Formula:C22H43NO CAS:112-84-5 MolWeight:337 RetIndex:2625
CompName:13-Docosenamide, (Z)- $$ Erucylamide $$ Erucyl amide $$ (Z)-13-Docosenamide $$ 13-Docosenamide, cis- $$ Armid E $$ cis-13-Docosenamide $

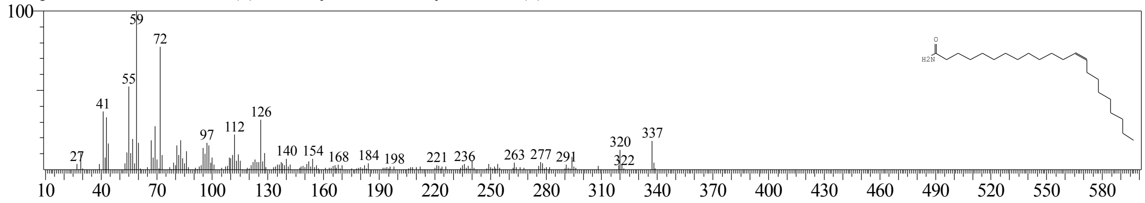

<< Target >>
Line#:32 R.Time:26.295(Scan#:4260) MassPeaks:376
RawMode:Averaged 26.290-26.300(4259-4261) BasePeak:59.05(125205)
BG Mode:Calc. from Peak Group 1 - Event 1

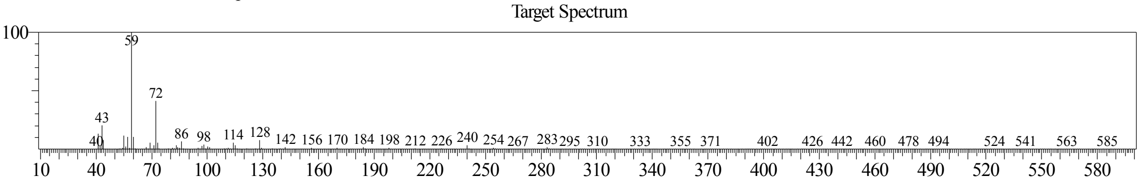

Hit#:1 Entry:105140 Library:NIST11.lib
SI:96 Formula:C18H37NO CAS:124-26-5 MolWeight:283 RetIndex:2220
CompName:Octadecanamide $$ Stearamide $$ Adogen 42 $$ Octadecamide $$ Octadecylamide $$ Stearic acid amide $$ Stearic amide $$ Stearoylamide $$ Stea

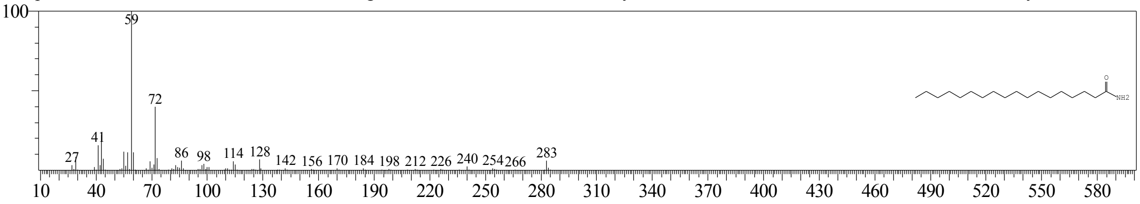

Hit#:2 Entry:62912 Library:NIST11.lib
SI:93 Formula:C14H29NO CAS:638-58-4 MolWeight:227 RetIndex:1822
CompName:Tetradecanamide $$ Myristamide $$ Myristic acid amide $$ Myristic amide $$ Tetradecylamide $$

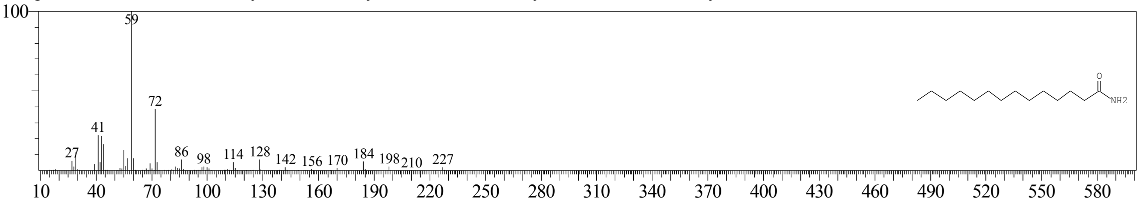

Hit#:3 Entry:271776 Library:Wiley9.lib
SI:93 Formula:C16H33NO CAS:629-54-9 MolWeight:255 RetIndex:0
CompName:Hexadecanamide (CAS) $$ Amide 16 $$ Amide HPL $$ Cetyl amide $$ Palmitamide $$ Palmityl amide $$ Palmitic amide $$ n-Hexadecanamide $$

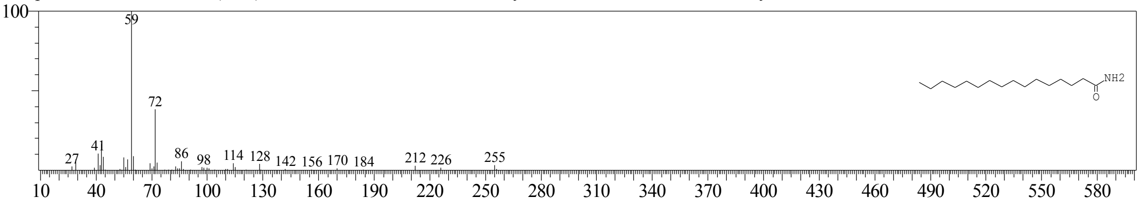

Hit#:4 Entry:83422 Library:NIST11.lib
SI:92 Formula:C16H33NO CAS:629-54-9 MolWeight:255 RetIndex:2021
CompName:Hexadecanamide $$ Palmitamide $$ n-Hexadecanamide $$ Amide HPL $$ Amide 16 $$ Cetyl amide $$ Palmitic acid amide $$ Palmitic amide $$ Pa

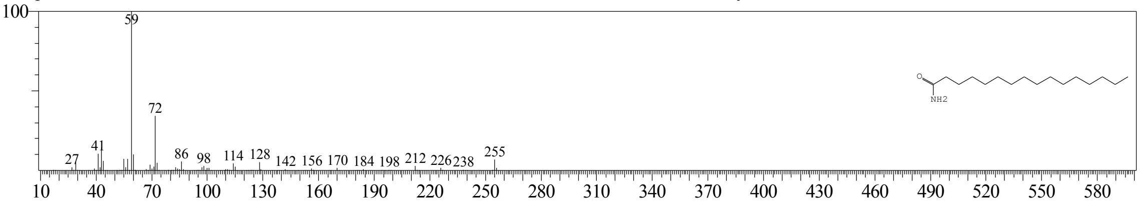

Hit#:5 Entry:43281 Library:NIST11.lib
SI:92 Formula:C12H25NO CAS:1120-16-7 MolWeight:199 RetIndex:1623
CompName:Dodecanamide $$ Lauramide $$ Amide KK $$ Dodecamide $$ Dodecylamide $$ Lauric amide $$ Lauryl amide $$ Lauroylamide $$ Diamide Y $$ n-

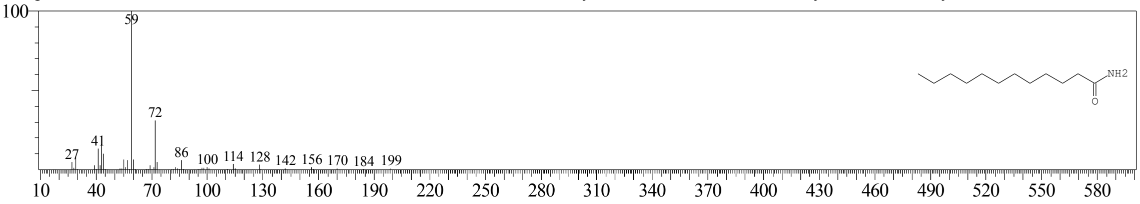

<< Target >>
Line#:33 R.Time:29.105(Scan#:4822) MassPeaks:358
RawMode:Averaged 29.100-29.110(4821-4823) BasePeak:59.05(22715)
BG Mode:Calc. from Peak Group 1 - Event 1

Target Spectrum

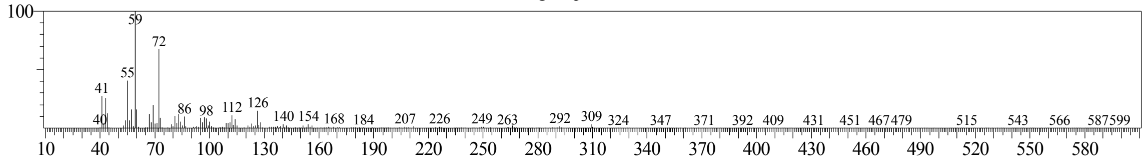

Hit#:1 Entry:332229 Library:Wiley9.lib
SI:94 Formula:C18H35NO CAS:3322-62-1 MolWeight:281 RetIndex:0
CompName:9-Octadecenamide (CAS) $$ 9-OCTADECENYLAMIDE $$ Armid ow $$ Armoslip cp $$ (9E)-9-Octadecenamide $$ 9 - octadecenamide $$ (9E)-9-

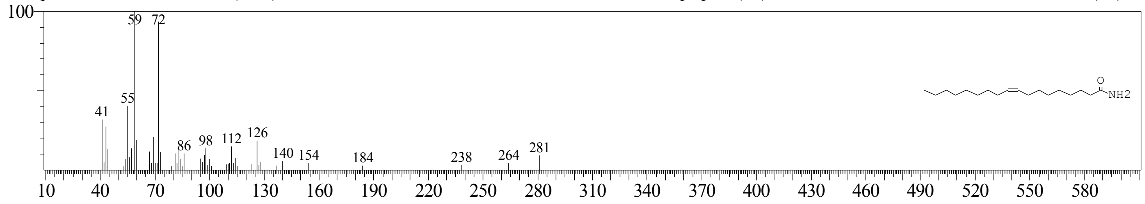

Hit#:2 Entry:332232 Library:Wiley9.lib
SI:94 Formula:C18H35NO CAS:301-02-0 MolWeight:281 RetIndex:0
CompName:9-OCTADECENAMIDE $$ 9-OCTADECENAMIDE, (Z)- $$ (9Z)-9-OCTADECENAMIDE # $$ (9Z)-9-OCTADECENAMIDE $$ (9Z)-9-OCTAD

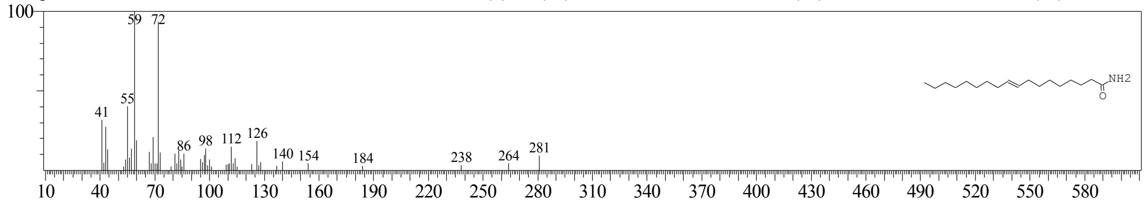

Hit#:3 Entry:332234 Library:Wiley9.lib
SI:90 Formula:C18H35NO CAS:301-02-0 MolWeight:281 RetIndex:0
CompName:9-Octadecenamide, (Z)- (CAS) $$ OLEOAMIDE $$ OELIC ACID AMIDE $$ Oleamide $$ Adogen 73 $$ Oleylamide $$ Slip-ezeCI) $$ Oleic acid a

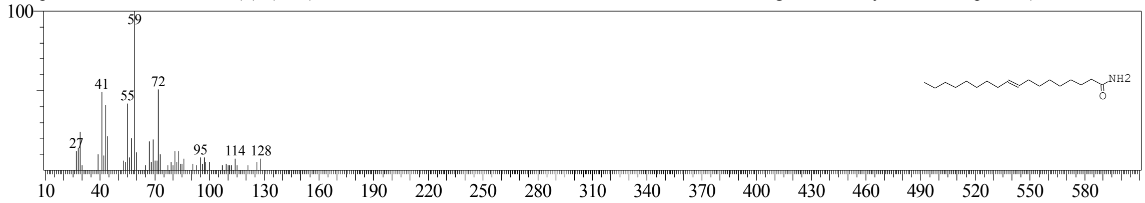

Hit#:4 Entry:103511 Library:NIST11.lib
SI:87 Formula:C18H35NO CAS:301-02-0 MolWeight:281 RetIndex:2228
CompName:9-Octadecenamide, (Z)- $$ Adogen 73 $$ Oleamide $$ Oleic acid amide $$ Oleyl amide $$ Slip-eze $$ Armoslip CP $$ Crodamide O $$ Crodamide

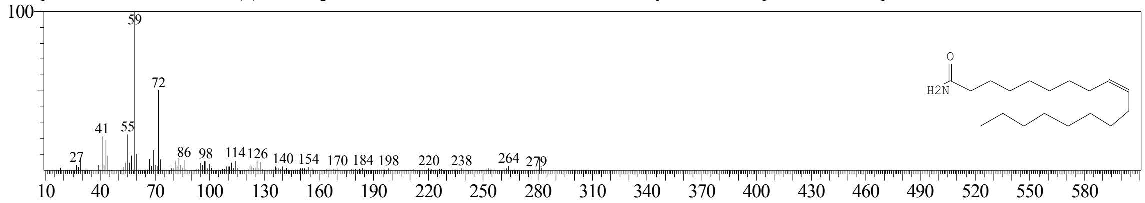

Hit#:5 Entry:146739 Library:NIST11.lib
SI:86 Formula:C22H43NO CAS:112-84-5 MolWeight:337 RetIndex:2625
CompName:13-Docosenamide, (Z)- $$ Erucylamide $$ Erucyl amide $$ (Z)-13-Docosenamide $$ 13-Docosenamide, cis- $$ Armid E $$ cis-13-Docosenamide $

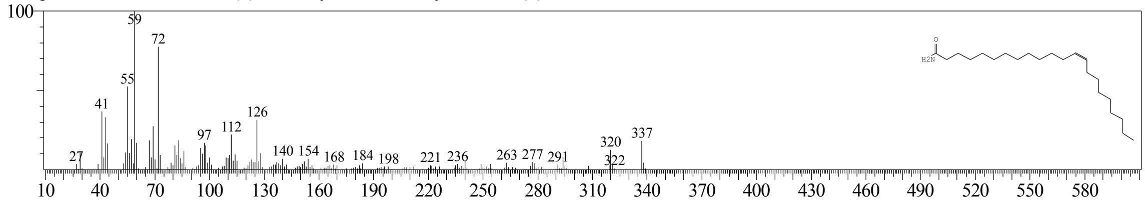

<< Target >>
Line#:35 R.Time:32.215(Scan#:5444) MassPeaks:427
RawMode:Averaged 32.210-32.220(5443-5445) BasePeak:59.05(223750)
BG Mode:Calc. from Peak Group 1 - Event 1

Target Spectrum

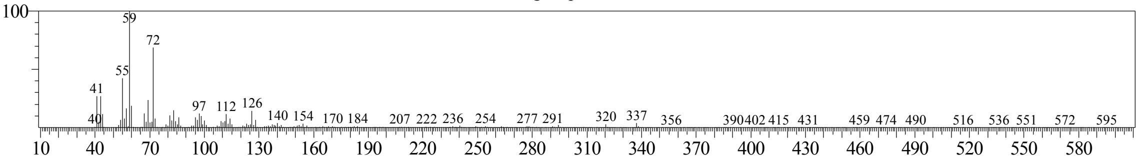

Hit#:1 Entry:332229 Library:Wiley9.lib
SI:94 Formula:C18H35NO CAS:3322-62-1 MolWeight:281 RetIndex:0
CompName:9-Octadecenamide (CAS) $$ 9-OCTADECENYLAMIDE $$ Armid ow $$ Armoslip cp $$ (9E)-9-Octadecenamide $$ 9 - octadecenamide $$ (9E)-9-

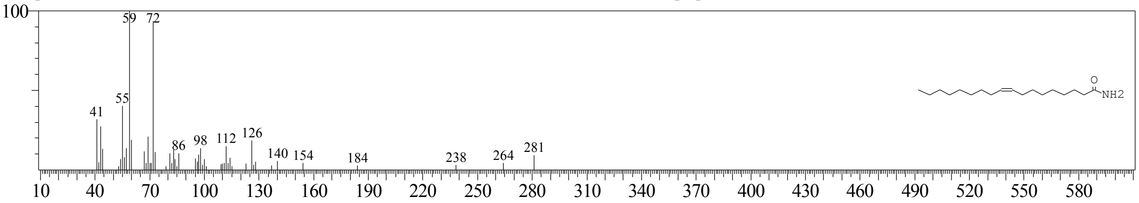

Hit#:2 Entry:332232 Library:Wiley9.lib
SI:94 Formula:C18H35NO CAS:301-02-0 MolWeight:281 RetIndex:0
CompName:9-OCTADECENAMIDE $$ 9-OCTADECENAMIDE, (Z)- $$ (9Z)-9-OCTADECENAMIDE # $$ (9Z)-9-OCTADECENAMIDE $$ (9Z)-9-OCTAD

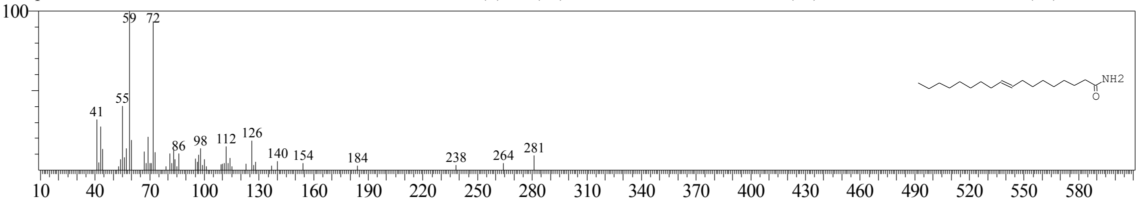

Hit#:3 Entry:146739 Library:NIST11.lib
SI:90 Formula:C22H43NO CAS:112-84-5 MolWeight:337 RetIndex:2625
CompName:13-Docosenamide, (Z)- $$ Erucylamide $$ Erucyl amide $$ (Z)-13-Docosenamide $$ 13-Docosenamide, cis- $$ Armid E $$ cis-13-Docosenamide $

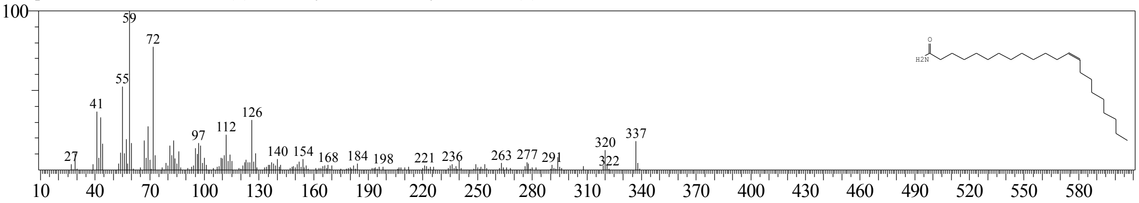

Hit#:4 Entry:332234 Library:Wiley9.lib
SI:88 Formula:C18H35NO CAS:301-02-0 MolWeight:281 RetIndex:0
CompName:9-Octadecenamide, (Z)- (CAS) $$ OLEOAMIDE $$ OELIC ACID AMIDE $$ Oleamide $$ Adogen 73 $$ Oleylamide $$ Slip-ezeCI) $$ Oleic acid a

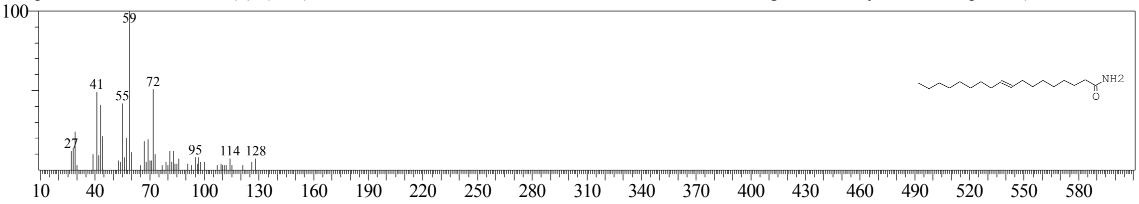

Hit#:5 Entry:103511 Library:NIST11.lib
SI:86 Formula:C18H35NO CAS:301-02-0 MolWeight:281 RetIndex:2228
CompName:9-Octadecenamide, (Z)- $$ Adogen 73 $$ Oleamide $$ Oleic acid amide $$ Oleyl amide $$ Slip-eze $$ Armoslip CP $$ Crodamide O $$ Crodamide

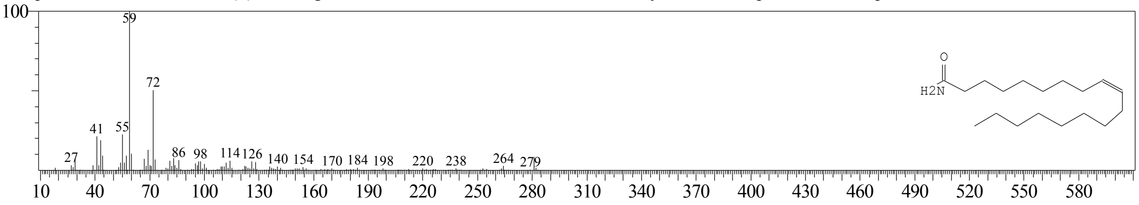

Supplement: Supplementary file 1 [file cimb-47-00401-s001.zip › cimb-3645934-supplementary.pdf]
